# Phased epigenomics and methylation inheritance in a historical *Vitis vinifera* hybrid

**DOI:** 10.1186/s13059-025-03858-2

**Published:** 2025-11-17

**Authors:** Noé Cochetel, Amanda M. Vondras, Rosa Figueroa-Balderas, Joel Liou, Paul Peluso, Dario Cantu

**Affiliations:** 1 Department of Viticulture and Enology, University of California Davis, Davis, CA USA; 2 Pacific Biosciences, Menlo Park, CA USA; 3 Genome Center, University of California Davis, Davis, CA USA

**Keywords:** Phased epigenomics, Sequence graph, Methylation inheritance

## Abstract

**Background:**

Epigenetic modifications, such as DNA methylation, regulate transcription and influence key biological traits. While many efforts were made to understand their stability in annual crops, their long-term persistence in clonally propagated plants remains poorly understood. Grapevine (*Vitis vinifera*) provides a unique model, with cultivars vegetatively propagated for centuries.

**Results:**

Here, we assemble the phased genomes of Cabernet Sauvignon and its parental lineages, Cabernet Franc and Sauvignon Blanc, using HiFi long-reads and a gene map tenfold denser than existing maps. Using three clones per cultivar, we quantify methylation with very consistent short- and long-read sequencing and ensure both varietal representativeness and assessment of clonal variability. We leverage the parent-progeny sequence graph to highlight allele-specific methylation and conserved transcriptomic patterns for genes and small RNA. Such a format is essential to integrate multi-omics data and reveals that, despite less clonal conservation than genetic polymorphisms, methylation marks are remarkably inherited. By further demonstrating the linear-reference limitations, we determine that the correct representation of genetic variants by the sequence graph is crucial for the accurate allelic quantification of the methylome.

**Conclusions:**

These findings reveal the remarkable stability of epigenetic marks in a model propagated by asexual reproduction. Using a phased sequence graph, we introduce a scalable framework that accounts for genomic variation, accurately quantifies allele-specific methylation, and supports multi-omics integration such as our evaluation of the transcriptional impact of epigenetic inheritance. This approach has broad implications for perennial crops, where epigenetic variation could influence traits relevant to breeding, adaptation, and long-term agricultural sustainability.

**Supplementary Information:**

The online version contains supplementary material available at 10.1186/s13059-025-03858-2.

## Background

Genetic and epigenetic marks shape genome function and regulate key biological processes, influencing traits such as development, stress responses, and adaptation [1]. Among these, DNA methylation plays a critical role in regulating gene and transposable element (TE) expression, impacting how organisms respond to their environment and maintain cellular identity across generations [2, 3]. In perennial plants, epigenetic modifications are particularly important as they affect phenotypes and plant adaptability over long lifespans [4]. Many fruit trees and other horticultural crops are propagated vegetatively to preserve desirable traits and ensure genetic and phenotypic consistency across generations. Without sexual reproduction to reshuffle genetic material, clones largely retain the genetic landscape of their progenitors, raising questions about the long-term stability of epigenetic information and how it evolves independently of genetic changes [5].

The accurate quantification of an individual’s methylome strongly depends on the concomitant availability of its genome sequence and the proper resolution of its ploidy at the haplotype level. Using a single reference genome is known to have a profound impact when characterizing genetic polymorphisms, and methylation analysis is the most affected [5]. By sequencing and phasing individual genomes, we can overcome the inherent biases of classic single-reference analyses, enabling a more accurate representation of polymorphisms, structural variants, gene content, and epigenetic marks. Beyond individual genome assemblies, pangenome graphs have emerged as a powerful approach to overcome the limitations of linear references, integrating multiple genomes into a unified framework that captures structural variation and epigenetic diversity [6]. The graphical format not only overcomes the single-reference bias but also integrates phasing information by storing phased haplotypes.

Grapevine (*Vitis vinifera*) has a long history of domestication, which involved transitions of reproductive system from the wild species, obligate outcrossers, to the hermaproditic cultivated grapes (*Vitis vinifera* spp. *vinifera*). Despite having the potential to self-pollinate, cultivation of domesticated grapes primarily relies on hybridization and clonal propagation to avoid inbreeding depression. The vegetative propagation of cultivars contributes to the fixation of desirable phenotypes by conserving highly heterozygous mutations beneficial to grape cultivation [7]. While genomic diversity has been characterized among grape clones, to what extent the epigenetic landscape is inherited during cultivar propagation remains largely unknown [8]. One of the most famous cultivars for winemaking, Cabernet Sauvignon, is derived from a cross between Cabernet Franc and Sauvignon Blanc. The hybridization event is suggested to have occurred spontaneously around the 17th century [9]. Such a model presents an outstanding opportunity to assess the persistence of parental genomic and epigenetic signatures in a well-established, clonally propagated lineage.

In this study, we assembled and annotated the genomes of Cabernet Sauvignon, Cabernet Franc, and Sauvignon Blanc to investigate to what extent methylation marks are conserved in a vegetatively propagated model. The varietal representativeness of the data relied on the usage of three independent clones as biological replicates, and the methylomes were supported by both short- and long-read sequencing. By accurately phasing the genomes using a high-density gene-based map, we compared Cabernet Sauvignon to its parental lineage haplotypes, enabling us to track allele-specific methylation, gene expression, and miRNA-mRNA networks. By applying a sequence graph approach to this trio, we not only provided new insights into the long-term stability of epigenetic inheritance in a clonally propagated hybrid but also overcame the bias inherent in single-reference approaches. Our findings not only advance the study of grapevine epigenomics but also offer a broader integrative framework via sequence graphs for investigating epigenetic conservation in genomic trios formed through hybrid crossings.

## Results

### Phased genome assembly and methylation quantification for the Cabernet Sauvignon parent-progeny trio

The Cabernet Sauvignon (CS) genome was assembled by combining HiFi sequencing data, a high-density gene map, and parental lineage information (Table 1). The gene map was developed using several *Vitis vinifera* genomes selected for the high contiguity of their assembly. About 20,000 genes were designated as highly conserved markers which represent a tenfold increase in density compared to the classic rhampseq markers [10] commonly used for scaffolding with HaploSync [11]. In addition to longer reads and a richer scaffolding map, sequencing data from Cabernet Franc (CF) and Sauvignon Blanc (SB) were also used to improve the phasing and assign a parent of origin to each haplotype (i.e., hapCF and hapSB instead of hap1/2). Moreover, new assemblies for CF and SB were generated using HiFi reads with the high-density gene map, resulting in significantly improved statistics when compared with previous assembly versions of the same cultivars [11, 12] (Table 1). The contig N50 for CS (20.1 Mb) was higher than CF (3.9 Mb) or SB (3.6 Mb) due to the use of a newer generation sequencing platform (Revio). The accuracy of the phasing was further supported by the clear separation of the CS haplotypes when aligned on CF and SB phased assemblies (Additional file 1: Figure S1A and S1B), also highlighting a balanced contribution of each parental chromosome (Additional file 1: Figure S1C).

**Table 1:** Assembly and annotation statistics for the three reference genomes

Cultivar	Cabernet Franc	Cabernet Sauvignon	Sauvignon Blanc
Clone	01	08	06
Size (Mb)*	474 | 461 | 64	491 | 487 | 37	474 | 458 | 46
Sequence count*	19 | 19 | 207	19 | 19 | 298	19 | 19 | 209
Contig N50	3.9	20.1	3.6
Gene count*	22,537 | 22,244 | 736	29,681 | 29,466 | 1558	23,659 | 23,106 | 730

*Values for haplotype 1, 2, and unplaced sequences, respectively. For CS, hapCF, hapSB, and unplaced are represented

We constructed whole-genome bisulfite sequencing (BS-Seq) libraries for CF, CS, and SB, each represented by three individual clones considered here as biological replicates (Additional file 2: Table S1). The overall average bisulfite conversion rate was 99% (Additional file 2: Table S2) for the three cultivars. The preparation of the genomes and the mapping were performed with bismark [13]. To prevent any reference bias, BS-Seq reads were mapped onto their corresponding reference genome (e.g., CS reads were mapped onto the CS assembly). High-quality sites were obtained by filtering out low-confidence cytosine sites (coverage < 10) and merging common sites present in the three clones for each cultivar. Methylation levels were also estimated directly from the HiFi reads for technical validation and showed strong support to the short-read sequencing data independently of the chemistry used (Pearson’s correlation *r* = 0.94, *P* < 2.2^e − 16^).

### Distribution of the methylation in the trio of phased genomes

**Fig. 1:**
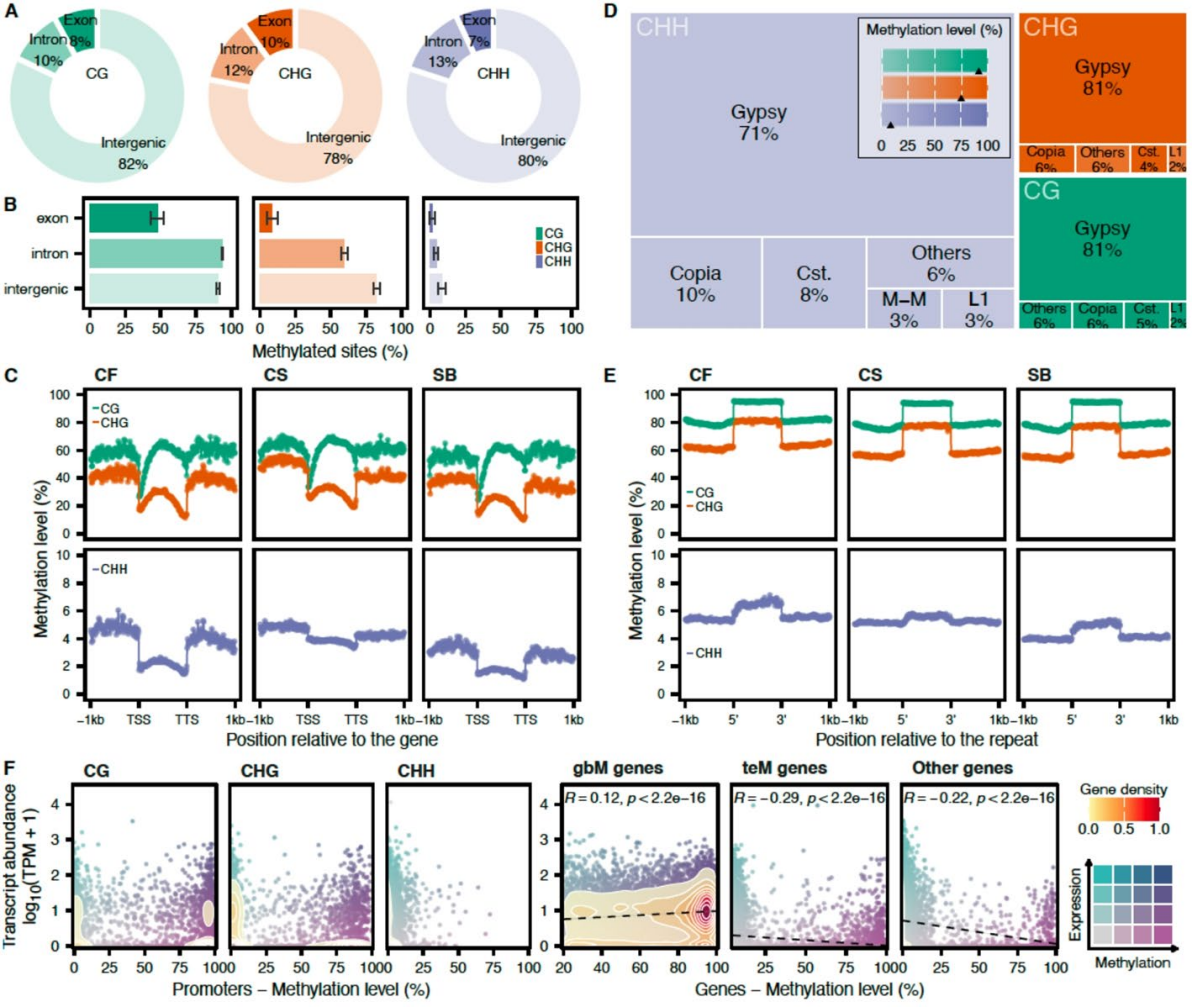
mCGs, the most abundant methylated cytosines, are negatively associated with gene expression except for gbM genes. **A** Distribution of the total number of cytosine contexts relative to the gene space (*n* = 9 clones). CS was used as a reference for intergenic space size normalization. **B** Proportion of methylated sites (methylation level > = 20%) per context group, mean ± sd (*n* = 9 clones). **C** Methylation level relative to gene loci. The points represent the average methylation values per bin (*n* = 3 clones). The − 1 kb upstream, the gene loci, and the + 1 kb downstream sequences were each divided into 100 bins. **D** Treemap of the total number of cytosine contexts in repetitive sequences. The transparency level represents the average methylation level per repeat class. In the legend key, the average methylation level per context is represented by a black arrow. **E **Methylation level relative to repeat sequences. The points represent the average methylation values per bin (*n* = 3 clones). The bins were obtained as described for the genes. CG, CHG, and CHH contexts are colored in panels A-E in green, orange, and purple, respectively. TSS: Transcription Start Site; TTS: Transcription Termination Site. Cst.: Custom, as defined by repeatmasker. **F** Gene expression relative to methylation level in promoter regions (3 kb upstream of the TSS) and in genes for the three cultivars. For genes, Pearson’s correlation coefficient and the associated *p*-value are represented at the top. For visual representation, points were randomly subsampled to 10,000. For gbM genes, only the CG context is represented. gbM: gene body methylation, teM: TE-like methylation

From the BS-seq data analyzed with the corresponding reference per cultivar, an overall range of 20–24.7.7 Mb total cytosine sites was considered among the different clones, accounting for a similar proportion of CG, CHG, and CHH contexts (Additional file 2: Table S3). About 73% of the sites were CHH contexts, while CG and CHG only represented 12 and 16% over the 19 chromosomes of CF, CS, and SB (Additional file 2: Table S3). Overall, the average methylation levels were similar among the three cultivars with a consistent CF > CS > SB order for each context (Additional file 2: Table S4). Independently of the methylation status, a comparable distribution within and outside the gene space was observed for the three contexts and the three cultivars, with about 80% in the intergenic space (76.2% − 83.3%, Additional file 2: Table S5), 11.9% in introns (9.6% − 14.27%, Additional file 2: Table S5), and 8% in exons (5.95% − 10.44%, Additional file 2: Table S5) (Fig. 1A). Taking into consideration the methylation level, methylated cytosines (presenting at least 20% of methylation) in CG (mCG) and CHG (mCHG) contexts were the most abundant, while CHH showed significantly lower methylation overall (Fig. 1B). For all the contexts, the highest percentage of methylated sites was detected in the intronic and intergenic regions. A remarkably high number was observed in the exons for the CG context. Relative to gene loci, a noticeable drop in methylation level was detected in the proximity of the transcription start site (TSS) in the three cultivars (Fig. 1C), followed by an increase reaching its highest point approximately in the middle of the genes, then decreasing toward the transcription termination site (TTS). Overall, CGs are the most methylated sites in genes, followed by CHG. The CHH levels were overall very low (Fig. 1C).

In repetitive sequences, CGs, CHGs, and CHHs were found to be distributed similarly with LTR/Gypsy and LTR/Copia being the predominant classes (Fig. 1D). Once again, the overall methylation levels were significantly higher in CG (91.4%) and CHG (74.9%), while only 8.6% was detected for the average methylation level in CHH. The levels detected in repeats were relatively higher when compared with the genes (Fig. 1E). The shift in methylation relative to the proximity to the repeat sequence was found to be very defined, with a noticeable jump in methylation level accounting for a 20% increase in average for CG and CHG sites (Fig. 1E). While being the most abundant context in the genome, CHH sites were characterized by a very low methylation level in the repeats.

The BS-Seq data were integrated with RNA-Seq data to evaluate the impact of methylation on gene expression. For CS, CG methylation in promoter regions (3 kb upstream of the TSS) (Fig. 1F) was mostly found in genes presenting very low or no expression (40.28% with TPM < 1) (Additional file 2: Table S6), while for CF and SB, mCGs were detected in a similar proportion in genes that were expressed or not (~ 30%). For the three cultivars, the lowest proportion of mCHGs was found in expressed genes. For CF and SB, most of the CHGs were not methylated and located in expressed genes. The context CHH was predominantly not methylated for the three genomes. For methylation occurring within gene loci, we observed a more defined impact on gene expression. Genes characterized by gene body methylation (gbM, genes methylated in CG only) were mostly expressed (Fig. 1F right panel). For genes with TE-like methylation (teM, genes presenting at least 5% of methylation for the three cytosine contexts) and the remaining (not falling into gbM nor teM categories), we observed a negative correlation overall (Fig. 1F right panel, Additional file 1: Figure S2). Taken together, these results highlighted that overall, the global methylome of each cultivar is comparable with characteristic methylation marks distributions and relation with gene expression.

### The trio pan-methylome revealed parental conservation of the methylation distribution in Cabernet Sauvignon

**Fig. 2:**
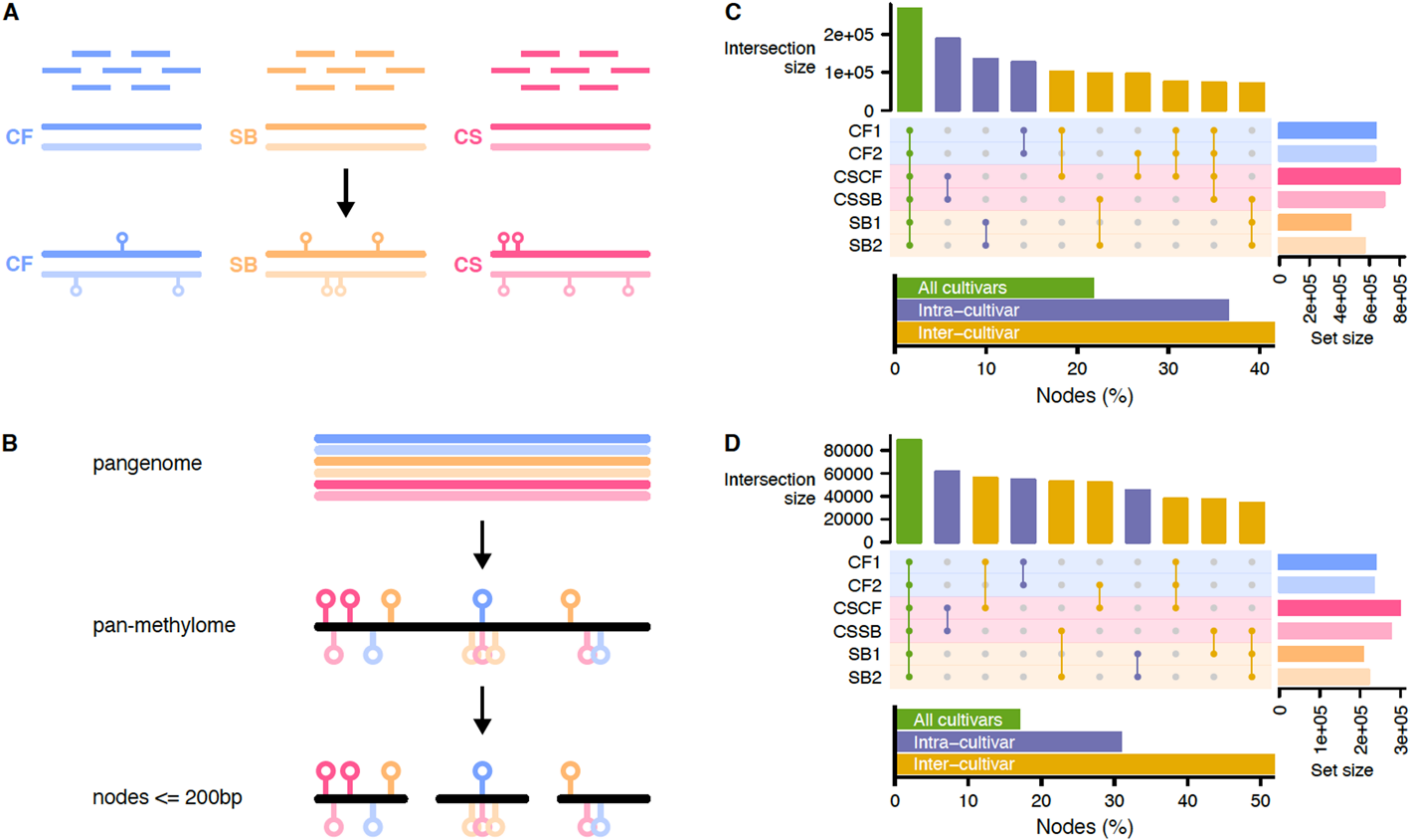
The pan-methylome captured inter-cultivar relatedness. **A** Cultivar-specific mapping of the bisulfite sequencing data. Each cultivar was represented by 3 individual clones. **B** Inferring the pan-methylome using a sequence graph. The cultivar-specific diploid methylation data were projected onto the graph. Nodes were chopped to be no longer than 200 bp. **C** Classification of the nodes containing cytosines. The upset plot represents the number of nodes for the top ten most represented combinations of haplotypes. Within these top ten combinations, the nodes proportion per cultivar class (all: node found in all haplotypes; intra: nodes found in the haplotypes of the same cultivar; inter: nodes in haplotypes of more than one cultivar) is represented on the bottom barplot. **D** Classification of the nodes containing methylated cytosines. The configuration of the plot is the same as in **C**. For **C** and **D**, CF1: Cabernet Franc haplotype 1, CF2: Cabernet Franc haplotype 2, CSCF: Cabernet Sauvignon haplotype CF, CSSB: Cabernet Sauvignon haplotype SB, SB1: Sauvignon Blanc haplotype 1, SB2: Sauvignon Blanc haplotype 2

Although analyzing the methylation results per genome prevented single-reference genome bias (Fig. 2A), performing comparative analyses among cultivars remains challenging. To overcome single-genome limitations, a sequence graph was built to represent each haplotype of the three cultivars with nf-core/pangenome [14, 15] (Fig. 2B). Within a graph, each haplotype is represented as a “path,” and paths are composed of sequences ranging from one to several base pairs, referred to as “nodes”. Windows were defined to compare methylation among the embedded paths (haplotypes) corresponding to nodes not longer than 200 bp, as defined as the optimal window size for the subsequent differential methylation analysis (DMA) (see Materials and Methods section for details). Nodes containing at least one cytosine context were then categorized into core (present in all haplotypes), dispensable (shared between two cultivars), and private class (only present in one cultivar), each category being exclusive.

Among the ten largest groups of nodes, the core nodes were the most abundant (Fig. 2C), followed by the nodes shared between the haplotypes of the same cultivar (private). Overall, the proportion of nodes detected within the same cultivar and between cultivars was similar. Considering the methylation status, we then defined nodes to be shared between haplotypes only if they presented the same methylation pattern. The methylation-aware distribution of the node groups was found to be enriched for inter-cultivar nodes (Fig. 2D). For CF and SB, more nodes were found to be shared with CS than between their own haplotypes (e.g., more methylated nodes between CF1 and CSCF than between CF1 and CF2). While many nodes were shared between haplotypes for CS (i.e., between CSCF and CSSB), the number of nodes shared with the parental haplotypes was proportionally significantly higher when methylation was considered. Altogether, the distribution of methylation patterns between CF and SB, and the phased haplotypes of CS suggest that part of its allele-specific methylome was explained by the methylation found in its parents. It is worth noting that among the combinations of shared nodes from the sequence graph, more than 96% of the shared sequences also present the same methylation status. A good example to confirm the potential inheritance of methylation patterns was to investigate the sex-determining region (SDR), which is known to be highly conserved at the genomic level [16]. Most of the methylated sites in the SDR were located between the *TREHALOSE-6-PHOSPHAT*E (*T6P*) and the *INAPERTURATE POLLEN 1* (*INP1*) encoding genes (Additional file 1: Figure S3), a region known to be very repetitive [17]. By evaluating the methylation patterns, we were able to infer unambiguously the parent of origin for each of the SDR alleles in CS. CSCF (Additional file 1: Figure S3B, left panel) was derived from CF hap1 (Additional file 1: Figure S3A, left panel), and CSSB (Additional file 1: Figure S3B, right panel) was inherited from SB hap2 (Additional file 1: Figure S3C, right panel). The analysis of this conserved genomic region clearly supports that the allele-specific methylation observed in the CS progeny was inherited from the parents.

### The differential methylation analysis in regions characterized by allele-specific methylation revealed an overall hyper-methylation pattern shared by CS and CF

**Fig. 3:**
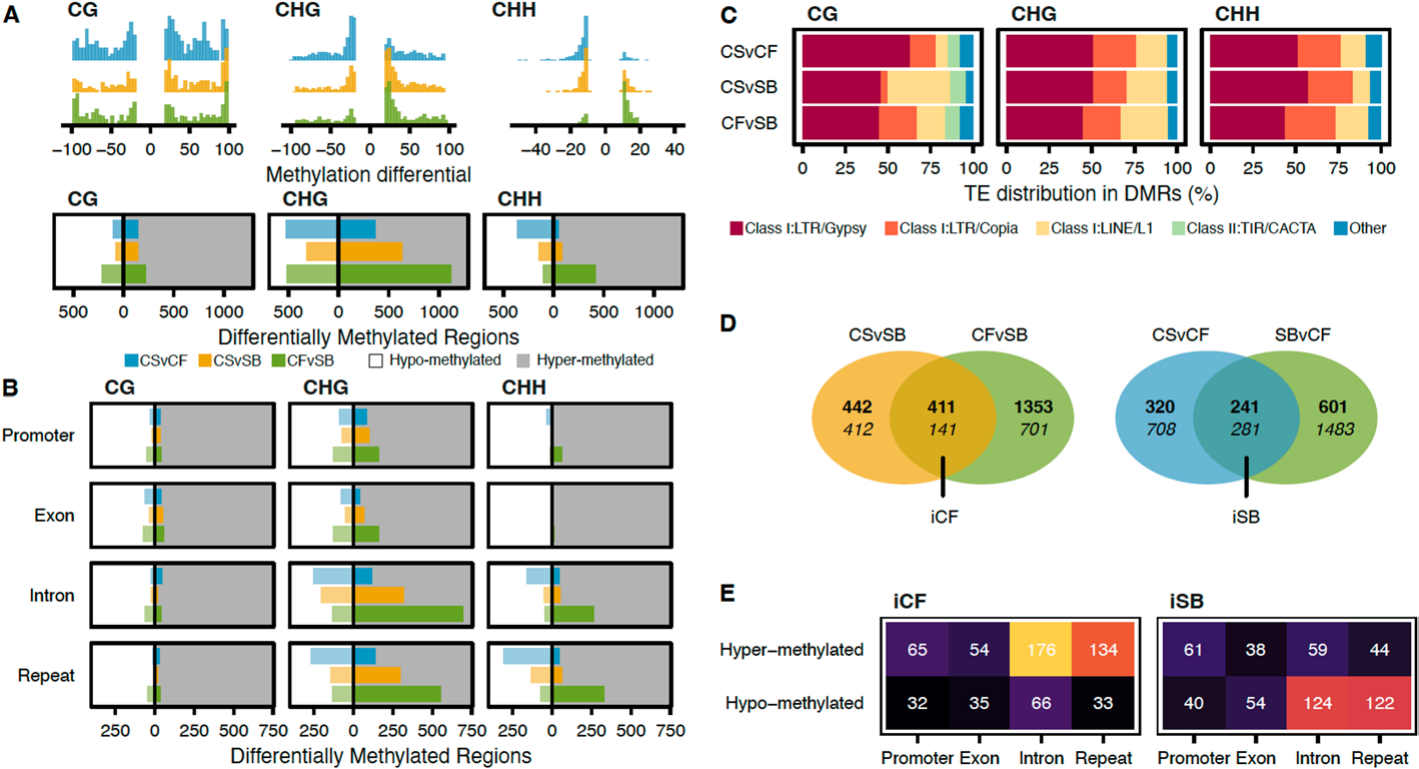
Patterns of methylation from parent lineages are conserved in CS. **A** Top panel, distribution of the methylation differential for each cytosine context and comparison (*n* = 50 bins); bottom panel, number of hypo- (white background)/hyper-methylated (grey background) regions. **B** Distribution of the differentially methylated regions (DMRs) per comparison in the gene space (promoter, exon, and intron) and the repeats. White and grey background colors represent hypo- and hyper-methylated regions, respectively. **C** Distribution of the transposable element (TE) content within the DMRs. The ‘Other’ category regroups all the remaining classes that represent less than 5% of the overall TE distribution. **D** Venn diagram representations of the differential methylation analyses and creation of the iCF and iSB subsets. The top numbers are hyper-methylated regions; the bottom numbers are hypo-methylated regions. **E** Distribution of the iCF and iSB DMRs in the genes and repeats

We leveraged the structure of the graph to perform differential methylation analysis (DMA) by extracting comparable regions among the embedded paths. By projecting 200 bp regions from CS onto the other haplotypes, the corresponding ranges of nodes were extracted and compared (Additional file 2: Table S7). To make sure we take into consideration the distribution of methylation levels per context and apply appropriate cutoffs, we performed a preliminary analysis and defined context-specific thresholds for the methylation differential (CG and CHG: 20%; CHH: 10%) (Additional file 1: Figure S4). Overall, DMRs in CG context were characterized by higher methylation differential values when compared to CHG; most of the methylation differentials for CHH were comprised in a lower range with absolute values between 10 and 20 (Fig. 3A, top panel). There were more differentially methylated regions (DMRs) when the parent cultivars were compared together (CF vs. SB; for any comparison hereafter the second genotype is considered as the reference, here SB) than with CS (Fig. 3A, bottom panel). There was a similar number of hyper-/hypo- DMRs in CG, while in CHG, an overall pattern of hyper-methylation was observed in CF whereas SB was characterized by an opposite pattern (hypo-methylation). For CHH, regions were mostly hyper-methylated in CF when compared to CS and SB (Fig. 3A, bottom panel). The distribution of the DMRs within the gene and repeat space tended to follow the general trend (Fig. 3B). Most of the DMRs were detected in introns and repeats (32.6% and 35.7% respectively). Interestingly, a high proportion of the significant DMRs located in introns contained transposable elements (TEs) for the context CHH (80.7% on average, Additional file 2: Table S8), followed by CHG (61.7%). Inversely, for the context CG, the DMRs were mostly located in introns not associated with TEs (27.2%, Additional file 2: Table S8). Very few DMRs were detected for CHH in promoters and exons (7.2% and 1.6% respectively). Among the repeats, the TE distribution was different among the comparisons, although it followed closely the overall genome composition (Fig. 3C, Additional file 2: Table S9). Less than 5% of the CG DMRs detected between CS and SB contained LTR/Copia TEs (4.55%). Instead, a higher abundance of LINE/L1 (36.4%) was detected for this comparison when compared to the two others. The distribution of TEs for the CHG and CHH DMRs was relatively similar, with the LTR/Gypsy being preponderant (~ 50%). The TIR/CACTA class was only found over 5% in the CG DMRs.

In order to assess whether the DMR patterns observed in CS can be attributed to a single parental cultivar, we compared the DMR sets and created two sets of inherited DMR (iDMR; DMR pattern observed in CS and only one of its parental cultivars). The iCF DMR set contains regions with the same differential for both CS vs. SB and CF vs. SB, while the iSB set contains DMRs found in common between CS vs. CF and SB vs. CF (Fig. 3D). Hyper-methylated iCF were about three times as much abundant as the hypo-methylated iDMRs (411 hyper-/141 hypo-methylated). On the other hand, iSB were characterized by a balanced differential (241/281 iDMRs). Interestingly, both iCF and iSB presented more hyper-methylated in promoter regions than hypo-methylated (Fig. 3E). The same pattern was observed for exons for iCF, while a higher number of hypo-methylated regions were detected for iSB. For introns and repeats, the pattern of the iDMRs demonstrated a clear pattern of allelic-specific methylation for the regions shared between CS and CF when compared to SB.

To further explore how the parental lines can impact the methylation in CS, we evaluated to which extent a differential allelic methylation observed in the parents would impact the methylation pattern in the hybrid (e.g., what is the methylation status in CSCF of a region that shows a methylation differential between CF hap1 and CF hap2). Using the coordinate system of the sequence graph, we were able to extract homologous regions from the haplotypes 1 and 2 of each parent relative to CS to perform a DMA (Additional file 2: Table S10). We identified 1,290 regions comparable with the previous DMA, and which presented a significant difference between hap1 and hap2 in CF or SB in total. We computed methylation deltas between each of the parental haplotypes and CS and observed that for 91.7% of the intra-cultivar DMRs, CS presented a similar methylation level to at least one of the parental haplotypes (delta < methylation differential threshold) (Additional file 1: Figure S5). Furthermore, when we integrated these results with our previous large-scale haplotype alignments that we performed to evaluate CS phasing (Additional file 1: Figure S1), we observed that most of the macro-alignments per chromosome (81.3%) corroborate with the similarity between methylation levels of the parental haplotype and CS. In other words, taking the example of the CS haplotype hapCF, if an alignment was detected on one of the two CF haplotypes, the methylation values in CS hapCF will be more similar to the pattern observed in this haplotype.

### Assessing the impact of genomic variants on methylation through linear and graph-based approaches

While allele-specific methylation can strictly be attributed to differences in methylation levels, it can also originate from underlying genomic variants. To evaluate the impact of variants on the DMA, we performed three distinct analyzes: (i) a methylation quantification was performed using a single genome as reference to estimate the potential biases of such an approach; (ii) the variations embedded in the sequence graph were extracted and their distribution within DMRs was evaluated; (iii) the graph was genotyped by mapping short-reads from each sample to assess the clonal variability and the impact of polymorphic sites on methylation differential.

**Fig. 4:**
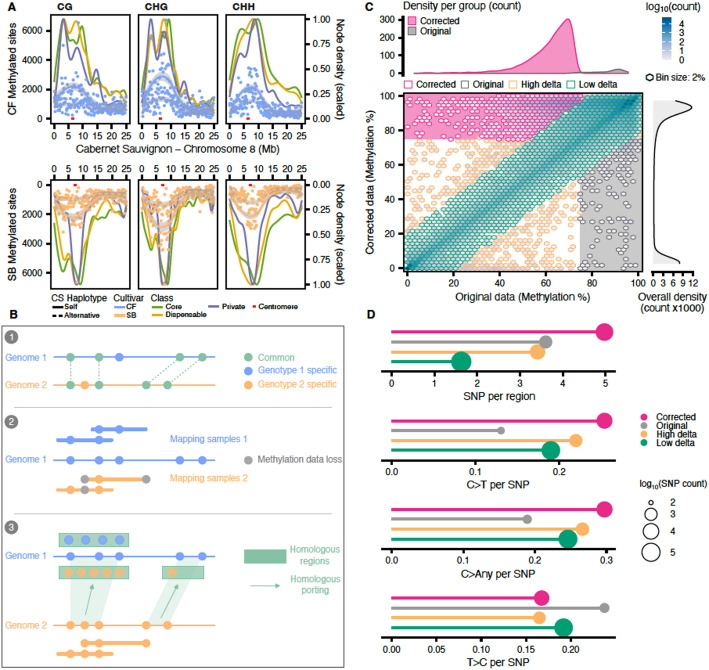
Methylation analysis using a single-genome reference is biased. **A** Distribution of the number of methylated sites per context for the CF (blue, top panel) and the SB (orange, bottom panel) samples. The chromosome 8 was selected as a representative example. The lines represent smoothed conditional means with a 0.95 confidence interval categorized into ‘self’ (solid line) when the sample was mapping on the corresponding CS haplotype (e.g., CF mapping on hapCF), and ‘alternative’ (dashed line) when the sample was mapping on the other haplotype (e.g., CF mapping on hapSB). For each plot, the three other curves represent the scaled distribution of the methylated nodes from the sequence graph. They were classed into core nodes if present in the three cultivars; dispensable nodes if present in CS and one of the parental cultivars; and private nodes if present only in either CF or SB. The red marker represents the location of the centromere. **B** Correction strategy using homozygous regions: there are genome-specific methylation sites (1) that are not represented when data from multiple genomes are mapped onto a single reference (2). After identifying homologous regions, methylation estimations can be corrected (3). **C** Comparison of the methylation levels before and after the homologous porting corrections. The CG context and the CSCF reference were selected as a representative example. On the main plot, each axis is divided into 50 bins. The frequency of regions detected within each bin is represented by the filling color gradient. Methylation deltas were calculated by comparing the average methylation level of a region before and after corrections. Bins were grouped into 4 categories: Corrected (pink color), data presenting at least 75% of methylation only post-correction and with a delta greater than or equal to 20%; Original (grey color), data presenting at least 75% of methylation only pre-correction and with a delta greater than or equal to 20%; Low delta (green color), data with a delta below 20%; and High delta (yellow color), data with a delta greater than or equal to 20%; each group is exclusive. The pink and the grey boxes cover regions where the methylation level is above 75% for the corrected and original data, respectively. The top density plot represents the frequency of regions in these boxes. The right density plot represents the overall density of the regions analyzed. **D** Distribution of polymorphisms and substitution types per group of regions defined in **C**. After extracting the variants between CSCF and CSSB, statistics were computed per group. Circle size is proportional to the number of SNPs

To evaluate the biases inherent to the use of a single reference, we restricted the BS-seq data analysis by mapping CF and SB samples onto the CS genome only. We observed a clear bias towards their corresponding haplotype (e.g., more methylation was detected when CF samples were mapped onto CSCF; Fig. 4A) which is partly explained by a higher mapping efficiency (Additional file 2: Table S11). Furthermore, we observed that the overall distribution of the methylated sites was consistent with the sequence graph and that the preferential mapping bias co-localizes with a peak of private and dispensable nodes (Fig. 4A). To explore whether underlying genetic variants can explain such a pattern, we had to produce an unbiased version of the mappings. To reach this goal, we designed a homology-based correction strategy. We defined homologous regions by sequence alignments and ported the corresponding methylation values (i.e., if we use CSCF as a reference, the mappings of SB samples onto CSSB are ported by homology to be represented on CSCF) (Fig. 4B). We classed the regions into four different groups labeled ‘corrected’ when the average methylation of the region reached a minimum of 75% and presented a high difference with the original estimations, and ‘original’ when the average methylation was high in the original data (75%) and presented a high difference with the corrected data (Fig. 4C). The threshold of 75% was set arbitrarily to select the regions with the most contrasted pre- and post-corrections for the subsequent polymorphism characterization. The other regions were classed into ‘low delta’ and ‘high delta’ based on the methylation difference before and after corrections, with a threshold set at 20%. By comparing the results before and after homology-based corrections, we highlighted a significant recovery of methylation levels (Fig. 4C). The alignments that were used to perform the homologous porting were then processed to extract polymorphisms between CS haplotypes and summarized for each group or region (Fig. 4D). We observed a striking enrichment for C > T substitutions inherently biasing the interpretation of differentially methylated cytosines (DMCs) by being undifferentiable from the bisulfite treatment (Fig. 4D). Overall, these results unambiguously confirmed that methylation level estimation strongly depends on the correct representation of the genomic diversity for the samples studied and that the use of a linear single reference is not optimal.

From the sequence graph, we extracted the embedded variants to evaluate how they impact the DMA results. For each region compared during the DMA, polymorphic sites for the pairwise comparison were considered (i.e., if the comparison is CF vs. SB, a site had to present a different genotype between CF and SB). Sites showing the same genotype were discarded, their presence originated from the graph deconstruction as it outputted variants for all the haplotypes. Variant sites were detected in a higher proportion of regions (84.3%) when the parent cultivars were compared (CF and SB, Additional file 2: Table S12), 10% higher on average than the comparisons involving CS (74.6%). The distribution of variant types was similar in the three cytosine sequence contexts, where single-nucleotide polymorphisms (SNPs) represented about 60% of the polymorphisms (Additional file 1: Figure S6). Among the substitution types inferred from the sequence graph, G > A and C > T accounted for almost 50% of the SNPs (Additional file 2: Table S13). No significant differences were observed for the distribution of substitution types within or outside DMRs. In other words, the graph-inferred regions defined for the DMA were not enriched for a specific polymorphism, which appeared to be the main limitation when identifying DMCs with the single-genome approach described above. Variants not categorized as SNPs, insertion-deletion (INDELs), or multiple-nucleotide polymorphisms (MNPs) were found to be the second most abundant (Other; 32%, Additional file 1: Figure S6). They represent the nested variation structures within the top-level variation bubbles reported by the variant projection from the graph. To assess the potential association between DMRs and structural variants, we extracted INDELs longer than or equal to 50 bp and calculated their distance relative to the DMRs. We observed that most of the short and large INDELs were in proximity (up to 1 kb) of CHG DMRs. Interestingly, the large deletions were characterized by a high proportion of variants occurring within the DMRs (Additional file 1: Figure S7A). By evaluating the proportion of DMRs that contain TEs, we observed that the average proportion of DMRs with overlaps was rather balanced (49% with overlaps, 51% without), but that the presence of TEs was enriched for the DMRs impacted by large deletions (Fisher’s Exact Test, *P* = 0.0018) (Additional file 1: Figure S7B). Altogether, these results suggest that large deletions preferentially affect TE-associated DMRs, highlighting an important role for this type of variant and transposable elements in the methylation dynamics differences observed between cultivars.

Going beyond the three genomes used to build the sequence graph, we considered the genetic variability of each clone by mapping DNA-Seq short reads onto the sequence graph and performed a variant genotyping. After normalizing and filtering the variant sites, we focused on the SNPs as they were the most abundant variant type and retained 3,095,637 sites. For CF and SB cultivars, most of the SNPs were identical among the three clones (Additional file 2: Table S14). Although a higher specificity was detected for the clone CS08 as it was the accession used as reference for variant representation, almost 70% of SNPs were common for the CS clones (Additional file 2: Table S15). Furthermore, a very similar number of sites were shared when pairwise comparing the clones of the two other cultivars (Additional file 2: Table S15), highlighting that despite one clone being selected as reference for the sequence graph construction, it did not impact the mapping or the variant detection for the others. Overall, SNPs were stable among clones, showing between 68.7 and 90.1% of similarity (Additional file 2: Table S15). For each cultivar, clones showed a lower conservation of the methylation sites, for which an average of 61% conservation was observed (Additional file 2: Table S3: Normalized/United sets). To understand whether these SNPs had a significant impact on the DMA, we analyzed the distribution of polymorphic sites within the windows that were pairwise compared. No differences were observed when comparing the SNP-containing regions to the overall distribution (Student’s t-test; *P* value > 0.05) (Additional file 2: Table S16). Furthermore, among the regions with polymorphic sites, no differences were observed in the number of SNPs for regions considered DMRs or not significant (Additional file 2: Table S17). Taken together, these results showed that the sequence graph was able to represent the clonal genomic diversity for each cultivar. The DMRs were not strictly explained by polymorphisms such as the C > T observed with the linear approach and rather represent regions with a true differential of methylation by incorporating genetic variations if there are any.

### Construction of a spliced pangenome graph to integrate transcriptomics with methylation

**Fig. 5:**
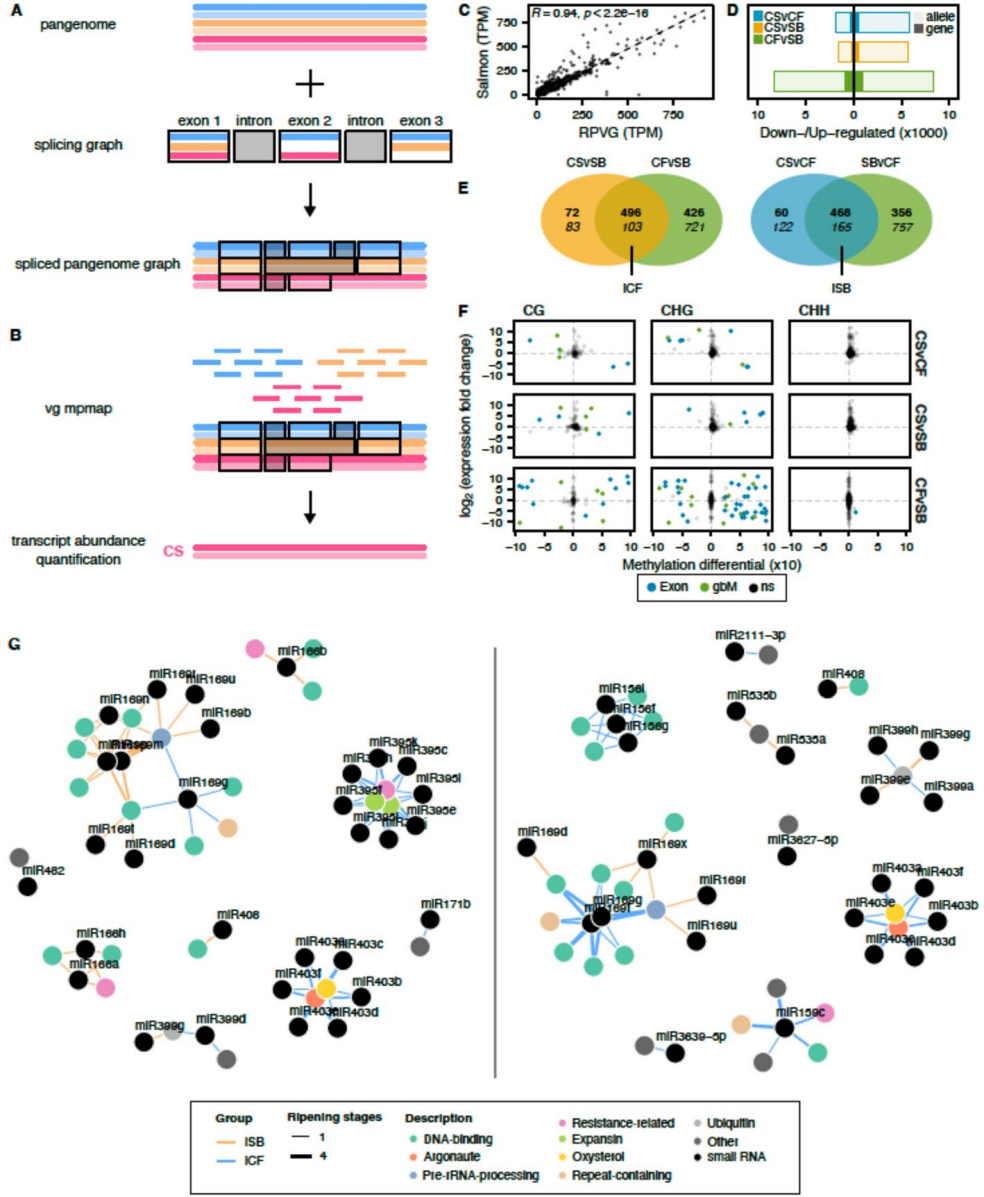
The spliced pangenome graph enables the integration of transcriptomics with methylation and small RNAs. **A** Creation of a spliced pangenome graph resulting from the combination of the sequence graph and a splicing graph. **B** Mapping of the RNA-Seq reads directly onto the spliced pangenome graph and transcript abundance quantification against a selected linear reference. **C** Correlation between transcript abundance quantified using Salmon and RPVG. **D** Number of differentially expressed genes per comparison. **E** Venn diagram representations of the differential expression analyses and creation of the iCF and iSB subsets. The top numbers are up-regulated genes, bottom numbers are down-regulated genes. **F** Comparison of the methylation differential to the expression fold changes per cytosine context and comparison in the exons. Exons present in gbM genes are colored in green, otherwise they are blue. ns: not significant. **G** miRNA-mRNA network for miRNA precursors (left) and mature miRNAs (right). Colored vertices (genes) and black vertices (miRNAs) are connected by colored edges (iSB: orange; iCF: blue). The edge thickness represents the frequency at which the pairs were detected among the different ripening stages

Methylation within or around gene loci can ultimately impact their expression. To integrate our transcriptomic data with the inferred pan-methylome, a spliced pangenome graph was constructed (Fig. 5A). By combining the gene exon-intron junctions of the three different cultivars and the sequence graph, reads can be directly mapped onto the splice pangenome graph, which recapitulates both the genomic variants and the transcriptomic splicing information [18] (Fig. 5B). We observed a great consistency between the genome-level RNA-Seq analysis and the graph-based results (Fig. 5C). Moreover, mapping on the sequence graph increased the mapping rate by 19% on average (Additional file 2: Table S18) as reads have a larger sequence repertoire to align to. Since the graph was built using phased genomes, allele-specific expression can be captured, which is especially important to consider in the case of a parent-hybrid trio analysis. To consider the presence of alleles and represent their expression at the gene level, we used two independent methodologies to identify allelic pairs in CS: (i) gene annotations from one haplotype (e.g., CSCF) were lifted over the alternative haplotype (e.g. CSSB) and vice versa; (ii) CDS from one haplotype were aligned on the other. By selecting reciprocal matches that were detected by the two methods, we identified allelic pairs and compared their expression. Overall, we were able to assign approximately 80% of the genes (46,831) from CSCF (23,420) and CSSB (23,411) to an allelic pair. The remaining genes fall into three categories: gene loci that were only detected unidirectionally (6,929), genes that did not overlap any gene annotation in the alternative haplotype (5,088), and genes that did not lift or map onto the other haplotype (299).

After performing a differential expression analysis (DEA), out of the 16,644 DEGs that were detected in the parental comparison CFvSB (Fig. 5D), 11,043 DEGs derived from an allelic pair (DE alleles). Almost half of the DE alleles (4,917) showed a mirrored expression with an up-regulation of the CSCF allele for the CF samples and reciprocally for the SB samples with the corresponding CSSB allele (Fig. 5D, Additional file 2: Table S19). When summarized at the gene level, the expression of each member of the same allelic pair generally cancels out each other, leading to a 9-fold lower absolute number of DEGs when compared to DE alleles, although the overall patterns remained conserved for CFvSB and the other comparisons (Fig. 5D). The other half of the DE alleles was generally characterized by a monoallelic regulation (Additional file 2: Table S19). As we were interested to know whether allele-specific expression was transmitted from only one parent (e.g. is CSCF allele in CF and CS more expressed than the CSSB allele from SB?), alleles were considered up-regulated when they were significantly higher expressed in their corresponding parent. In other words, in a CFvSB comparison, the allele CSCF was considered up-regulated in CF if it was an up-regulated DEG while the allele CSSB was considered up-regulated in SB if it was a down-regulated DEG from the same comparison. Interestingly, when looking at the comparisons involving CS, CSvCF and CSvSB, up-regulated genes were resulting from either an up-regulation of both alleles or an up-regulation of only one parental allele (e.g. the allele CSSB was up-regulated in both CSvCF and CFvSB comparisons) (Additional file 2: Table S20). By comparing the DEG sets, we were able to identify genes with an inherited expression pattern from only one of the two parents, named hereafter iCF for a similar expression between CS and CF, and iSB for a conserved expression between CS and SB (Fig. 5E). Within iCF and iSB, we focused on genes with an assigned allelic pair and observed two patterns (Additional file 1: Figure S8): (i) for iCF up-regulated genes (Additional file 1: Figure S8A), when the CSCF allele is highly expressed in CF, it presented an intermediate expression level in CS and a low expression in SB, however, the pattern was not conserved for the CSSB allele (the same was observed for the iSB set in Additional file 1: Figure S8B); (ii) genes presenting a down-regulation pattern (more expressed in SB in the case of the iCF set, Additional file 1: Figure S8A; more expressed in CF in the case of the iSB set, Additional file 1: Figure S8B) were characterized by a conservation of their expression pattern in both alleles (CSCF and CSSB) suggesting an important cis regulation to repress their transcription.

By comparing the DMA results to the DEA performed at allele level, three noticeable characteristics were observed: (i) DMRs in exons (Fig. 5F) were associated with a higher differential for gene expression overall when compared to DMRs occurring in promoters (Additional file 1: Figure S9), (ii) for the CHH context, DEGs were detected almost exclusively for DMRs occurring in promoter regions (Additional file 1: Figure S9), (iii) the set of differentially methylated and expressed genes was enriched in gbM genes (Fisher’s Exact Test, *P* < 2.2e-16) (Fig. 5F).

To further explore the potential conservation of transcriptomics signatures inherited from CF or SB in CS, we integrated our graph-based results with small RNA (sRNA) sequencing of the same leaf samples. We observed a typical distribution with 24 nt as the most abundant size in the leaves (Additional file 1: Figure S10A). The 21-nt-long small interfering (si)RNAs were located within exons, while the 24-nt were found in repetitive regions and promoters (Additional file 1: Figure S10B). The number of micro (mi)RNA clusters detected with this approach was very limited (92). Among the roles attributed to siRNAs, their activity has been previously documented to regulate methylation [19]. We performed a DEA and identified 2,852 differentially expressed sRNA clusters (DE sRNA clusters). The genomic location of the 24-nt-long DE sRNA clusters was compared with the previously identified DMRs, and 12 clusters were considered being located within 1 kb of the DMRs. Interestingly, 11 of these 12 DE sRNA clusters also showed inherited differential of expression between CS and CF or SB (as defined previously for the iCF and iSB DEG sets) (Additional file 2: Table S21), but this pattern was not concomitantly observed for the methylation.

Since the first approach is not designed for the miRNA class detection, we supplemented it with another analysis relying on the pipeline nf-core/smallrnaseq [20] and the database miRbase 22.1 [21] to quantify miRNA precursors and mature miRNAs. We also expanded the experimental design to include berry samples at different ripening stages for the three cultivars. After performing DEA for both genes (Additional file 1: Figure S11A) and miRNAs (Additional file 1: Figure S11B-C), we compared the sets of DEGs and differentially expressed miRNAs (DEmiRs) to identify iDEGs and iDEmiRs (i.e., genes or miRNAs that have a common expression differential between CS and only one of the two parental genotypes). On average, 76% of the DEmiRs had a least one of their targeted genes concurrently differentially expressed. The number of iDEGs and iDEmiRs was then compared and classed by expression differential (up-/down-regulated) (Additional file 1: Figure S11D-E). Interestingly, for iCF, most miRNA-mRNA pairs showed a negative relationship (i.e., if the miRNA is down-regulated, the targeted gene was found up-regulated) while for iSB, the proportion of positive and negative pairs was more balanced (Additional file 1: Figure S11F). By building a miRNA-mRNA network for iCF and iSB negative pairs, for the miRNA precursors (Fig. 5G, left panel) and the mature miRNAs (Fig. 5G, right panel), we observed inherited patterns. Most of the targeted genes were involved in DNA-binding functions, mostly transcription factors. Some miRNA precursors were only detected for iCF, with for example, different forms of miR395 and miR403 targeting the same genes involved in biotic stress resistance, argonaute, and expansin-related functions. The miR169 network was the most abundant in targeted iDEGs for both iCF and iSB; however, for mature miRNAs, the network was significantly more stable across berry ripening stages in CF and CS (iCF). Also, the miR403 miRNA-mRNA network was detected exclusively for iCF in the mature miRNAs. Altogether, this network analysis revealed that numerous miRNA-mRNA negative pairs co-exist in CS, while they were exclusively detected in CF or SB.

## Discussion

This study demonstrates that despite centuries of clonal propagation, epigenetic marks can be conserved to a certain extent, long after the original parental inheritance. Since cultivars undergoing many cycles of vegetative propagation can accumulate somaclonal epimutations [22, 23], each of the cultivars was represented here by three distinct clones.

Methylation is known to be one of the most sensitive omics to the choice of the reference [5]. Single-reference approaches introduce several layers of bias, from impeding the read mapping to limiting the analysis to reference-specific methylation sites and gene annotations. Such a strategy ultimately impairs the proper differential methylation analysis and makes it nearly impossible to distinguish allele-specific patterns. Comparing methylation regions between distinct genomes was challenging, but the correct phasing of the assemblies and the use of the graphical format overcame these limitations. We have not only demonstrated that regions can be defined within a sequence graph to compare methylation across haplotypes (Fig. 2B) but also confirmed that the use of a single-reference genome led to strongly biased results (Fig. 4) as reported in previous work using a coordinate mapping between divergent genotypes [24]. The sequence graph generated in this study represented the clonal variations for each cultivar by genotyping the embedded polymorphisms. However, simplifying the complexity of such a graph could provide a better representativeness of the clonal variability by incorporating the new polymorphisms detected after mapping, but it remains computationally challenging [6]. Our results also showed that by corroborating results from short-read data, the direct detection of methylation from long-read sequencing allows the acquisition of high-quality methylomes in parallel with whole genome sequencing with no additional cost [25].

A higher number of genes methylated in CG and CHG contexts, showing low or no expression, was observed for the cultivar CS (Additional file 2: Table S6), averaging around 10% more than CF or SB relative to their total gene contents. Hybrids often show non-additive methylation patterns – the progeny’s methylome is not just an average of the parents. Methylation can be instead reinforced, especially in regulatory regions, suggesting epigenetic dominance or methylation gain in hybrids [26, 27]. Such a pattern was independently supported by the sequence graph with CS having the highest number of private methylated nodes (Fig. 2D). Interestingly, while a high similarity was expected for the intra-cultivar nodes (shared between the haplotypes of the same cultivar genome, Fig. 2C), the shared methylated regions were more numerous between the corresponding parent-progeny haplotypes, displaying an enrichment for the inter-cultivar nodes when methylation status was considered. Independent of sequence differences, these methylation patterns supported that a parental signature was conserved within the progeny methylome.

To further explore to what extent we could detect allele-specific methylation, we performed many differential analyses to compare the parental lineages and the hybrid (iCF and iSB, Figs. 3D and 5E). A very stable result was observed throughout our analyses. The most variable cytosine context, over-represented in the iCF and iSB sets, was CHG and mostly occurred in repetitive regions, particularly in TEs (Fig. 3). Non-CG methylation is commonly found near transposable elements [1] making it a good candidate for varietal epigenetic differences. In our comparisons, such a signature was characterized by a hyper-methylation of the CHG iDMRs for CS and CF when compared with SB (Fig. 3E), but also by its significant association with SVs such as large deletions (Additional file 1: Figure S7). The methylation status of CGs was more stable among the genomes (Fig. 3A and B); however, a few DMRs in TIR/CATCA DNA transposons were specifically detected for this context (Fig. 3C). Furthermore, CG DMR overlapping *Copia* LTR retrotransposons occurred more frequently when CF was involved in the comparison. While involved in a significantly lower number of DMRs, these results do not exclude the important contribution of the mCGs in allele-specific methylation. On the other side, methylation was almost undetectable in the CHH context for the three cultivars (Fig. 1). The configuration process of the DMA parameters reflected this pattern (Additional file 1: Figure S4) with an optimal threshold set twice as low (10%) as for the other cytosine contexts (20%). The overall low CHH methylation at the genome level was observed previously in grapes and other plant species [3, 28], and is generally expected in clonally propagated species [29].

Overall, our sequence graph strategy helped us to mitigate the biases of reference-based methods. We further expanded its usage by integrating splicing information and performed a pantranscriptomic analysis [18] (Fig. 5A and B). The stronger methylation differential observed for CHG (Fig. 3) was associated with a higher number of DEGs when gene loci had CHG DMRs in their exons or promoter regions (Fig. 5F). By investigating the groups of DEGs-DMRs (Fig. 5F), we observed that their relationship was not strictly correlated and more nuanced than when we compared expression levels directly to methylation levels (Fig. 1F). The correlation between methylation and expression is known to be influenced by many factors, including the precise position of the methylation marks within the genic regions [30] (Fig. 1C), but also the proximity to repetitive sequences, notably transposable elements (Fig. 1E) [31]. Furthermore, the CS transcriptome was found to express more genes (at higher levels) than CF and SB (Fig. 5D). The majority of the DEGs observed when CF and SB were compared were also shared with CS, suggesting that the allele showing higher expression was retained (Tables S19 and S20). Such a pattern of allele-specific expression was observed previously in other plant models and leads to non-additive gene expression patterns within hybrids [32]. The pantranscriptome was further integrated with small RNA-Seq data (Fig. 5G). In addition to DNA methylation, post-transcriptional regulation by sRNAs can indeed play a critical role in modulating gene expression. Our analysis of miRNA–mRNA expression pairs revealed that the sequence graph was able to capture that CS inherited distinct post-transcriptional regulation patterns from each parent. These parent-specific miRNA interactions may have contributed to the differential expression of target genes in CS and reflect regulatory divergence beyond direct transcriptional or epigenetic control.

## Conclusions

We demonstrated here that a sequence graph provides a powerful framework for studying methylation inheritance in clonally propagated species like grapes and is relevant for species far beyond the *Vitis* genus. By integrating genomic variation from phased genomes, the sequence graph allowed for the representation of both common and rare alleles, including structural variants and splicing information, both crucial for multi-omics integration to understand epigenetic regulation. Such an approach revealed the parental contributions to epigenetic modifications in their progeny, providing insights into the stability and heritability of methylation marks in a model hybridized about 300 years ago.

## Methods

### DNA sequencing

HiFi sequencing libraries for CS08 were prepared following the manufacturer’s protocol for the SMRTbell Express Template Prep Kit 3.0 (Pacific Biosciences, CA, USA) from young leaf tissue. Libraries were size-selected using the BluePippin system (Sage Science, Beverly, MA, USA) with a cut-off range of 10–50 kb. After size selection, the HiFi libraries were cleaned up using 1X (v/v) AMPure PB beads (Pacific Biosciences, CA, USA). The concentration and final size distribution of the libraries were evaluated using the Qubit 1X dsDNA HS Assay Kit (Thermo Fisher Scientific, Waltham, MA, USA) and the Femto Pulse System (Agilent, Santa Clara, CA, USA), respectively. The HiFi library was sequenced on a Revio sequencer at the DNA Technology Core Facility, University of California, Davis, CA, USA. For the HiFi libraries of CF01 and SB06, they were prepared as described previously [33] and sequenced using the Sequel II system. For the additional sequencing of CS clones (CS06, CS08, and CS47), a newer library preparation method was used in combination with the PacBio Revio SPRQ Chemistry. Before shearing, the ~ 3 µg of gDNA for each clone was run through Short Read Eliminator cleanup on the Hamilton Microlab Prep system following the manufacturer-recommended automation protocol. This was followed by shearing with the Megarupter 3 DNA shearing cartridge (Diagenode: Cat. No. E07010003). Samples were sheared at 30ng/ul with Speed 29 to achieve an insert size distribution ranging from 17 kb to 19 kb across the clonal gDNA samples. Following shearing, SMRTbell^®^ libraries were constructed for each sample and size selected using the dilute AMPure PB beads as previously described [34]. Samples were simultaneously sequenced with three independent runs in parallel on the Revio Sequencing System with the SPRQ polymerase (PacBio, PN:103–520-100) and SPRQ Sequencing Chemistry (PacBio, PN:103504-900). HiFi reads with methylation calls were generated on the instrument before downstream tertiary analysis. For the short-read sequencing, libraries and sequencing were performed as described previously [17].

### High-density gene map construction

The CDSs from genes annotated on PN40024 [35] were mapped separately on the haplotypes of selected genomes from grapegenomics.com [36]. Assemblies were selected based on their high contiguity from PacBio HiFi sequencing. The mapping was performed with three tools: GMAP v2019-09–12 [37], BLAT v36 × 2 [38], and minimap2 v2.17-r941 [39]. Thresholds of coverage and identity, maximizing the number of uniquely mapping genes, were identified separately for each mapper. For GMAP and minimap, the highest number of uniquely mapping genes was obtained by setting a threshold of at least 80% identity and coverage, while for BLAT, the minimum threshold had to be increased to 97%. The datasets were intersected to obtain a list of 20,165 gene models common to all methods. The alignment coordinates were compared across the selected haplotypes, and the CDSs with consistent order and location across all assemblies were retained. The final gene map consists of 19,049 markers based on the synteny of uniquely mapping gene models.

### Genome assembly and annotation

The genome assemblies of Cabernet Sauvignon clone 08 (CS08), Cabernet Franc clone 01 (CF01), and Sauvignon Blanc clone 06 (SB06) were generated from HiFi reads (Yield: 73.21Gb, 14.04Gb, and 12.27Gb respectively) as described previously [33], by combining the hifiasm outputs [40] with the high-density gene map produced in the present study using HaploSync [11]. The phasing of CS08 was further processed with the DNA sequencing data from the parental lineages to generate the haplotypes CF (hapCF) and SB (hapSB). The genome annotation of CS08 was performed as described in previous work [17]. For Cabernet Franc and Sauvignon Blanc, published annotations of Cabernet Franc clone 04 (CF04) [11] and Sauvignon Blanc clone 01 (SB01) [12] were lifted onto the newly assembled clones CF01 and SB06, respectively, using LiftOn [41] with the parameters “-a 0.95 -s 0.95”. The resulting lifted genes were filtered to only retain models coding proteins presenting 100% identity with the source models using diamond [42] blastp and “--id 100 --query-cover 100 --subject-cover 100”. Centromeres were annotated using the tool centIER v.2.0 [43].

### Whole-genome alignments

Each CS haplotype (hapCF and hapSB) was aligned against the four haplotypes from CF and SB (i.e., a multifasta with CF hap1, CF hap2, SB hap1, and SB hap2) using nucmer from the tool mummer v4.0.0rc1 [44]. Results were filtered to keep only the best alignments with ‘delta-filter − 1’. Coordinates were obtained using show-coords with the parameters ‘-c -l -T’. A last filter was applied to only retain 5-kb alignments with at least 90% identity.

### Total RNA and small RNA sequencing

Total RNA isolation was done using a Cetyltrimethyl Ammonium Bromide (CTAB)-based protocol as described previously [45] from young leaf tissue and whole deseeded berries (skin and pulp). Small RNA was purified from total RNA using the quick RNA miniprep kit (Zymo Research, Irvine, USA) as per the manufacturer’s protocol. RNA and Small RNA concentration and purity were assessed with Qubit (Life Technologies, Carlsbad, CA, USA) and a NanoDrop 2000c Spectrophotometer (Thermo Fisher Scientific, Waltham, MA, USA), respectively. RNA sequencing was performed as described previously [17]. Briefly, library preparation was performed with the Illumina TruSeq RNA sample preparation kit v.2 (Illumina, CA, USA) following the low-throughput protocol. After evaluation of library quantity and quality, the sequencing was performed using an Illumina HiSeq 4000 (DNA Technology Core Facility, University of California, Davis, CA, USA) to produce 100 bp-long and 150 bp-long paired-end reads for the leaves. For the berries, 100 bp-long single-end and paired-end reads were generated. Small RNA libraries were prepared using the Illumina TruSeq small RNA library prep kit (Illumina, CA, USA). A hundred nanograms of purified small RNA was used as starting material and 3’ and 5’ RNA adapters were ligated before the reverse transcription. During the PCR amplification of cDNA constructs, individual barcodes were added (with a total of 16 cycles). The libraries were then purified with 1.6X KAPA pure beads (Roche Diagnostics) and size selected with the BluePippin instrument (Sage Science, Beverly, MA, USA) using a 3% agarose gel cassette with a cut-off range of 120–170 bp. Size-selected libraries were cleaned with 1X KAPA pure beads (Roche Diagnostics, Mannheim, Germany). Libraries were quantified with Qubit (Life Technologies, Carlsbad, CA, USA) and validated with the Agilent High sensitive kit (Agilent Technologies, CA, USA), then pooled at equimolar bases. Leaf and the berry libraries were sequenced with 100 bp and 50 bp single-end on a HiSeq4000, respectively (DNA Technology Core Facility, University of California, Davis). To mitigate low mapping rates of the CS samples, a second round of sequencing was performed for all the leaf samples with 150 bp paired-end by IDSeq on a HiSeqX platform.

### Whole genome bisulfite sequencing (WGBS)

The EZ DNA Methylation Gold kit (Zymo Research, Irvine, USA) was used for sodium bisulfite conversion of DNA from young leaf tissue before library preparation. Each DNA sample was spiked with 0.5% unmethylated lambda DNA as an internal control. All WGBS libraries were prepared from 100 ng of HMW genomic DNA using a TruSeq DNA Methylation kit (Illumina, CA, USA) according to the manufacturer’s protocol. Paired-end sequencing (2 × 150 bp) was performed on a HiSeq4000 (DNA Technology Core Facility, University of California, Davis).

### Bisulfite sequencing data analysis

Pre- and post-trimming quality controls were performed using FastQC v0.11.8 [46] and summarized with MultiQC v1.10.1 [47]. Samples with 150-bp-long reads were trimmed down to 100 bp using trim_galore v0.6.10 [48] and the parameter “--hardtrim5 100”. Trimmed reads were further filtered for quality with the same tool using the parameters “--quality 20 --length 80”. Read depth was normalized across samples by down-sampling the read libraries to the lowest observed value (82462299, Additional file 2: Table S1) using seqtk v1.3-r106 (https://github.com/lh3/seqtk) with the flags “sample -s100”. After mapping the reads onto the lambda genome using Bismark [13] and parameters “--bowtie2 -N 1”, a bisulfite conversion > = 98% was observed for all the samples (Additional file 2: Table S2).

When the BS-seq reads were mapped onto the phased genome of their corresponding cultivar, e.g., CS reads mapped onto the CS genome (Additional file 2: Table S22), the parameters “--ambiguous --ambig_bam” were added. Any reads giving an ambiguous mapping (multi-mapping) and absent from the resulting alignment files were further mapped per haplotype. The low rate of residual ambiguous mapping after this second round supports that those reads mostly originated from homozygous regions (Additional file 2: Table S23). The alignments on the phased genomes were separated per haplotype to merge them with the corresponding ambiguous read alignments. Another set of alignments was generated by mapping all the samples on the CS genome only to assess single-reference bias.

Deduplication was performed using deduplicate_bismark from the bismark tool suite (Additional file 2: Table S24). Methylation statistics were extracted from the deduplicated files using bismark_methylation_extractor. After assessing methylation bias, the final methylation extraction was performed using “bismark_methylation_extractor --paired-end --gzip --multicore 24 --buffer_size 12G --CX_context --cytosine_report --comprehensive --bedGraph --ignore 15 --ignore_r2 15 --ignore_3prime 2 --ignore_3prime_r2 2 --output $extractor_outdir --genome_folder $genome_outdir $bam_output” (Additional file 2: Table S25).

### Methylation analysis at the genome level

Methylation data were processed using the R package methylkit v1.29.1 [49] in RStudio running R v4.3.3 [50]. Files were opened using methRead with “mincov = 0”, filtered using filterByCoverage and “lo.count = 10”, normalized with normalizeCoverage, and concatenated into a single file using unite and the parameter “destrand = FALSE” (Additional file 2: Table S3). For the intra-cultivar DMA (Additional file 1: Figure S5), one unite file was generated per parental cultivar following the same steps. After converting the unite objects and the structural annotations of each genome into Granges [51], the relative position of the methylation sites was determined using the function findOverlaps. To compare the distribution of cytosine contexts within and outside gene loci among genomes (Fig. 1A and Additional file 2: Table S5), the intergenic space size was normalized using the CS genome assembly as the reference. This step was performed to make the cytosine distributions comparable between a *de novo* genome annotation (CS) and the annotations derived from a lift over (CF and SB). The regions surrounding gene loci and repeats were binned to average the methylation levels. The upstream 1 kb, the gene/repeat loci, and the downstream 1 kb were each divided into 100 bins. Genes were categorized as gbM (gene body methylation) if their average exonic CG methylation was greater than or equal to 20% and lower than this threshold for CHG and CHH. Gene were classed as teM (TE-like methylation) if their average methylation was greater than or equal to 5% for all the cytosine contexts and not previously classed as gbM.

### Evaluation of the single-reference genome bias

Since more cytosines were detected by mapping the samples (CF or SB) onto their corresponding CS haplotype (hapCF or hapSB, respectively, Fig. 4A), the strategy was to define homologous regions so that methylation values could be ported. Only one haplotype can be considered by methylkit for the analysis. For example, if the haplotype hapCF is used, more cytosines will be detected for the CF samples (Fig. 4B). To correct this bias, results from the mapping of the SB samples on hapSB were ported from pre-defined homologous regions (Fig. 4B). Homologous regions were identified by comparing the CS haplotypes hapCF and hapSB using nucmer from mummer v4.0.0rc1 [44]. Each macro-region matching between both haplotypes was then cut into 200 bp chunks and mapped a second time using minimap2 [39]. After discarding any ambiguous mapping, mappings of the CF samples on hapSB and mappings of the SB samples on hapCF were corrected. Polymorphisms in homologous regions were extracted using the function delta2vcf from mummer (Fig. 4D).

### Optimization of the parameters for the differential methylation analysis

Pairwise comparisons were designed using the function “reorganize” from methylkit. Windows were defined using tileMethylCounts with “win.size” and “step.size” being 100-bp increments from 100 to 2000 bp, and “cov.bases” ranging from 1 to 10 (Additional file 1: Figure S4). The differential methylation analysis (DMA) was performed for each comparison using calculateDiffMeth and ‘overdispersion=“MN”’. Significant DMRs were defined using a qvalue < 0.01 and tested for an absolute differential > = 5, 10, 15, 20, and 25 (Additional file 1: Figure S4). DMRs were also identified using DSS [52]. The function DMLtest was used with smoothing = TRUE and the significant DMRs were extracted via callDMR using parameters with fixed values “p.threshold = 1e-5, minCG = 3”, variable delta values ranging from 0.05 to 0.25 by 0.05, and variable region sizes ranging from 50 to 1000 bp (Additional file 1: Figure S4). DMRs identified using methylkit were tested for overlaps by using the function reduce from GenomicRanges [51].

### Transcript abundance quantification at the genome level

Reads were trimmed based on quality using trimgalore [48] and the following parameters “trim_galore --quality 20 --length 80 --fastqc --gzip --fastqc_args \“-o fastqc/filtered\“ --paired $read1 $read2 --output_dir seq/filtered” (Additional file 2: Table S26). Salmon [53] was used to index (kmer size = 31) and quantify transcript abundance. For the three cultivars, each set of reads was mapped onto the corresponding diploid genome. Transcript abundance was summarized at the gene level using tximport [54]. The differential expression analysis was performed using DESeq2 [55].

### Sequence graph construction and variant genotyping

A sequence graph was constructed using the nf-core/pangenome pipeline [14, 15] which mostly relies on PGGB [56]. The six haplotypes were considered as distinct genomes (2 haplotypes x 3 cultivars) to construct 19 chromosome-level pangenomes with the following configuration: ‘“wfmash_map_pct_id”: 90, “wfmash_segment_length”: 10000, “wfmash_n_mappings”: 5’. Before mapping on the graph, DNA-Seq short reads were trimmed to not be longer than 100 bp using cutadapt v.1.18 [57] and “--length 100 --pair-filter both”. Three high-coverage samples (CF04, CS08, and SB01) were down-sampled to the average coverage of the six other samples (53.15 M of reads) using seqtk as described for the BS-seq data. The graph was converted to a vcf with vg deconstruct using CS haplotypes as the reference. Genetic variants were categorized using bcftools v.1.21 [58] (Additional file 1: Figure S6). Structural variants (SVs) were considered as INDELs with a length greater than or equal to 50 bp (Additional file 1: Figure S7). The relative positions of the variants to the DMRs were obtained using the function findOverlaps from GenomicRanges [51] with the parameter ‘maxgap = 1000’. The same vcf was used to construct a new graph using vg construct “-m 1024”. The xg index and the snarls were computed using vg index and vg snarls, respectively. After pruning the graph with vg prune and the parameter ‘-r’, a gcsa index was constructed with vg index. The paired-end reads were mapped with vg map, the read support was extracted using vg pack, and finally, the genotyping was performed with vg call and the option “-a”. After merging the resulting vcf of each sample with bcftools merge, the multi-sample vcf was filtered with bcftools filter “-e ‘MAF < = 0.25 || QUAL < 30 || INFO/DP < 10 || INFO/DP > 500’”. The vcf records were then normalized with bcftools norm “--atom-overlaps ‘*’ -m-any” and SNPs were extracted with bcftools view “-v snps”. A second round of bcftools filter was performed as described above before the final site annotation with bcftools annotate. Any duplicated sites due to the conversion of MNVs into SNVs were discarded. The final filtered vcf contained 3,095,637 SNPs.

### Pan-methylome inference and pan-transcriptome analysis

Before categorizing nodes, the sequence graph was chopped into nodes not longer than 200 bp with odgi chop [59]. To perform differential methylation analysis among cultivars, 200 bp windows were defined in the phased genome of CS (CSCF and CSSB). Odgi position [59] was then used to determine the corresponding regions in the other genomes. The matching ranges were further filtered to be 150–250 bp long and to contain at least 3 cytosines. A spliced pangenome graph was generated using vg [18]. The pggb sequence graph was converted to PackedGraph format with vg convert and chopped to contain nodes not longer than 32 bp using vg mod. A non-conflicting id space between the 19 pangenomes was obtained with vg ids before merging them all using vg combine. The spliced pangenome graph was finally generated using vg rna. Before mapping RNA-Seq reads, the gcsa and dist indexes were generated using vg index. The mapping was performed using vg mpmap using CS as a reference for gene expression. Counts were extracted from the multipath alignments using rpvg [18]. The differential expression analysis was performed following the genome-level pipeline described above.

### Annotating gene allelic pairs in the phased CS genome

To identify the corresponding allelic pairs between hapCF and hapSB in the CS gene annotation, two approaches were used. The gene annotations from each CS haplotype were lifted over the other haplotype using LiftOn [41] and the parameter ‘-exclude_partial’. To avoid any misplacement due to the absence of the unplaced sequences, we included them in the target sequences. The resulting lifted annotation files were filtered to retain mRNAs and overlapped with the native annotations from the target sequence using bedtools intersect v.2.30.0 and the parameters ‘-wa -wb -f 0.5’ [60] (i.e., overlapping the lifted hapCF annotations in hapSB with the actual hapSB annotations). The second approach consisted of the alignment of the CDS of each haplotype onto the other haplotype using GMAP v. 2019-09-12 [37]. The resulting mappings were filtered to only retain hits with at least 50% identity and coverage and overlapped with the native annotations as described above with bedtools intersect. For the two approaches, allelic pairs were only considered if a match between two genes (one from hapCF and one from hapSB) was reciprocal (detected in both directions of the analysis). Using the pairing information, the allele-level gene expression was summarized per gene before performing the differential expression analysis following the aforementioned protocol.

### Small RNA analysis

For the leaf samples, the two rounds of sequencing were concatenated by combining the single-end reads and the read 1 from the paired-end reads. Small RNA sequencing data were aligned and annotated using Shortstack v.4.1.1 [61] with the option ‘--autotrim’ (Additional file 2: Table S27). The read depth was extracted from the trimmed and condensed read fasta files. The annotated clusters were filtered to only retain valid DicerCalls. To characterize the miRNA, the nf-core/smallrnaseq pipeline [20] and miRbase 22.1 [21] were used. Reads were automatically trimmed during the process with fastp [62] (Tables S28 and S29). Counts were processed with DESeq2 [55] for differential expression analysis as described for the classic RNA-Seq analysis. The location of the significantly differentially expressed sRNAs was compared with the previously identified DMRs using GenomicRanges with the parameter ‘maxgap = 1000’.

## Supplementary Information

Additional file 1. Figure S1. Phasing of the Cabernet Sauvignon genome. Figure S2. Gene expression relative to methylation level per cytosine context. Figure S3. Methylation patterns are inherited at the sex-determining region. Figure S4. Benchmark for differentially methylated regions (DMRs). Figure S5. Methylation levels in the intra-cultivar DMRs. Figure S6. Distribution of the graph-inferred variants in the DMRs. Figure S7. Distribution of the variant types relative to their distance from DMRs. Figure S8. Allelic expression of the iCF and iSB gene sets. Figure S9. Gene expression relative to promoter methylation level per cytosine context. Figure S10. Distribution of the sRNAs in the genome of CS. Figure S11. Identification of iDEGs and iDEmiRs in berries. 

Additional file 2: table S1. Whole-Genome Bisulfite Sequencing read statistics (number of reads). Table S2. Bisulfite conversion statistics (%). Table S3. Methylkit data processing (number of cytosines). Table S4. Overall average methylation levels (%). Table S5. Cytosine contexts distribution (%). Table S6. Methylation vs. gene expression. Table S7. DMA results (number of significant hypo-/hyper-methylated regions). Table S8. Proportion of DMRs located in introns containing TEs (%). Table S9. TE composition of the Cabernet Sauvignon genome. Table S10. Intra-cultivar DMA results (number of significant hypo-/hyper-methylated regions). Table S11. Number of mapped reads after bismark mapping on each CS haplotype. Table S12. Number of windows with variants. Table S13. Substitution type distribution. Table S14. Number of SNPs shared between pairs of clones. Table S15. Number of SNPs shared between clones. Table S16. DMRs with polymorphic sites compared to the overall DMRs. Table S17. Average number of SNPs in regions considered DMR or not significant. Table S18. RNA-Seq mapping rates. Table S19. DEA results for the DE alleles. Table S20. Intersection of the DE alleles between the comparisons involving CS and the parental comparison CFvSB. Table S21. Differentially expressed sRNA clusters located within 1 kb of the DMRs and considered inherited (iCF/iSB). Table S22. Bismark results - mapping on phased genomes (number of reads). Table S23. Bismark results - mapping per haplotype (number of reads). Table S24. Bismark deduplication results (number of alignments). Table S25. Bismark results. Table S26. RNA-Seq read filtering. Table S27. Read statistics pos-trimming by ShortStack. Table S28. Read statistics pre- and post-trimming by fastp for the berry samples. Table S29. Read statistics pre- and post-trimming by fastp for the leaf samples.

## Data Availability

All the raw sequences were deposited on NCBI SRA under the BioProject number PRJNA1244791 [63]. The genome assemblies of CS, CF, and SB have been deposited in the European Nucleotide Archive (ENA) at EMBL-EBI under accession number PRJEB101820 [64]. Assemblies and annotations are available on Zenodo (CS, 10.5281/zenodo.15191822 [65]; CF, 10.5281/zenodo.15191856 [66], and SB, 10.5281/zenodo.15191869 [67]) and hosted on grapegenomics.com [36] with blast and jbrowse2 services. The matches from the LiftOn results between the previous and the new annotations were uploaded in the corresponding repositories (10.5281/zenodo.15191856 [66] and 10.5281/zenodo.15191869 [67]). Previous genome annotations for CF04 [11] and SB01 [12] were retrieved from grapegenomics.com [36]. The high-density gene map was uploaded in the Zenodo repository 10.5281/zenodo.15191822 [65]. The code and scripts used for the analyses are available on GitHub under MIT License ([https://github.com/noecochetel/Epigenomics_Trio]) [68] and on Zenodo [69].
