# Supplementary material for: Phased epigenomics and methylation inheritance in a historical Vitis vinifera hybrid
Source: Genome Biol. 2025 Nov 17;26:392. doi: 10.1186/s13059-025-03858-2 (PMC12621364; doi:10.1186/s13059-025-03858-2)
Supplement: Supplementary file 1 — Additional file 1. Figure S1. Phasing of the Cabernet Sauvignon genome. Figure S2. Gene expression relative to methylation level per cytosine context. Figure S3. Methylation patterns are inherited at the sex-determining region. Figure S4. Benchmark for differentially methylated regions (DMRs). Figure S5. Methylation levels in the intra-cultivar DMRs. Figure S6. Distribution of the graph-inferred variants in the DMRs. Figure S7. Distribution of the variant types relative to their distance from DMRs. Figure S8. Allelic expression of the iCF and iSB gene sets. Figure S9. Gene expression relative to promoter methylation level per cytosine context. Figure S10. Distribution of the sRNAs in the genome of CS. Figure S11. Identification of iDEGs and iDEmiRs in berries. [file 13059_2025_3858_MOESM1_ESM.pdf]

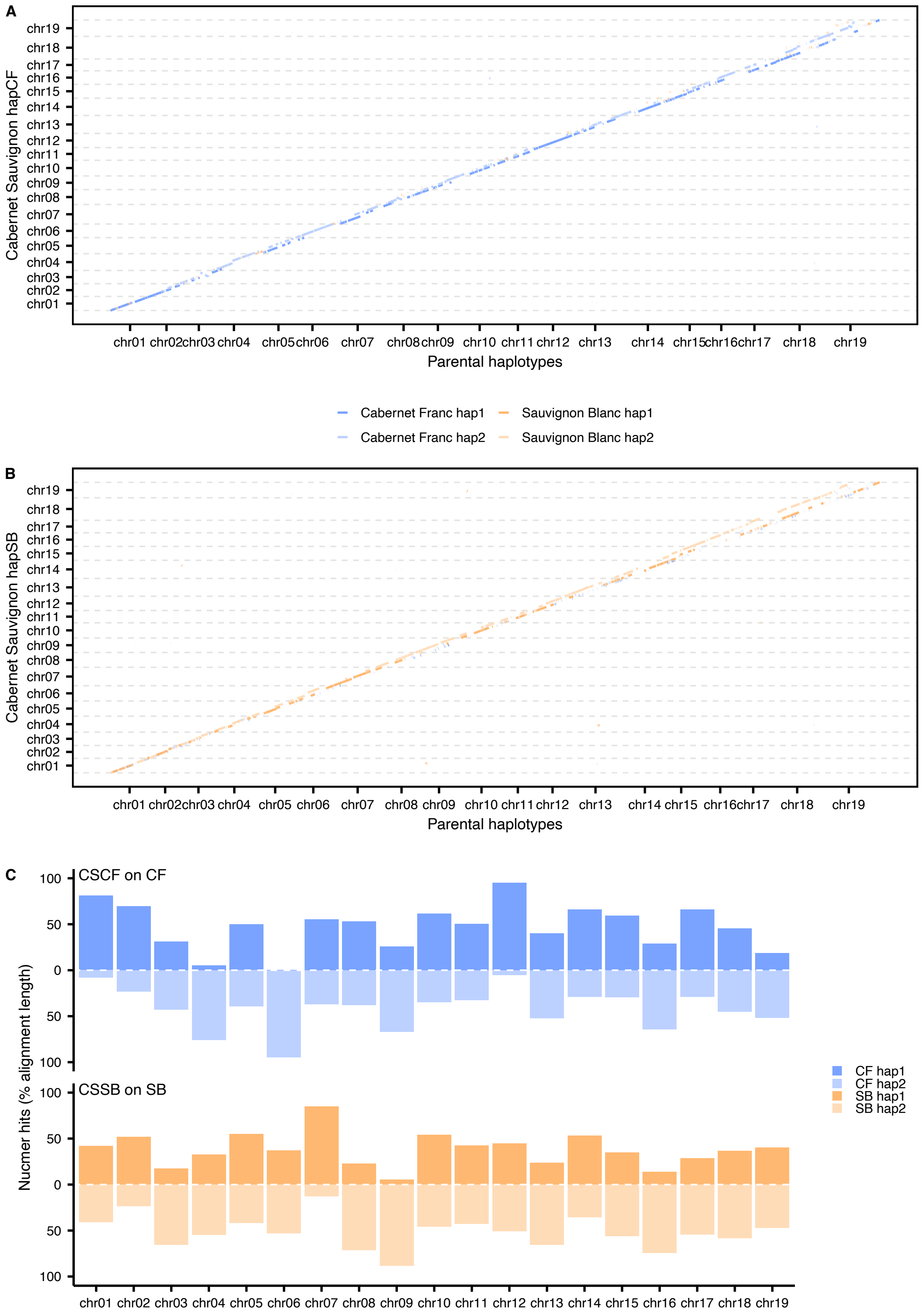

Figure S1. Phasing of the Cabernet Sauvignon genome.

After aligning each CS haplotype (**A,** hapCF and **B,** hapSB) simultaneously on the parental genomes (CF1, CF2, SB1, and SB2), nucmer best matches were filtered to only keep 5-kb-long hits with 90% identity. **C.** Distribution of the nucmer hits relative to the CS haplotype chromosome length represented as a percentage of the alignment length.

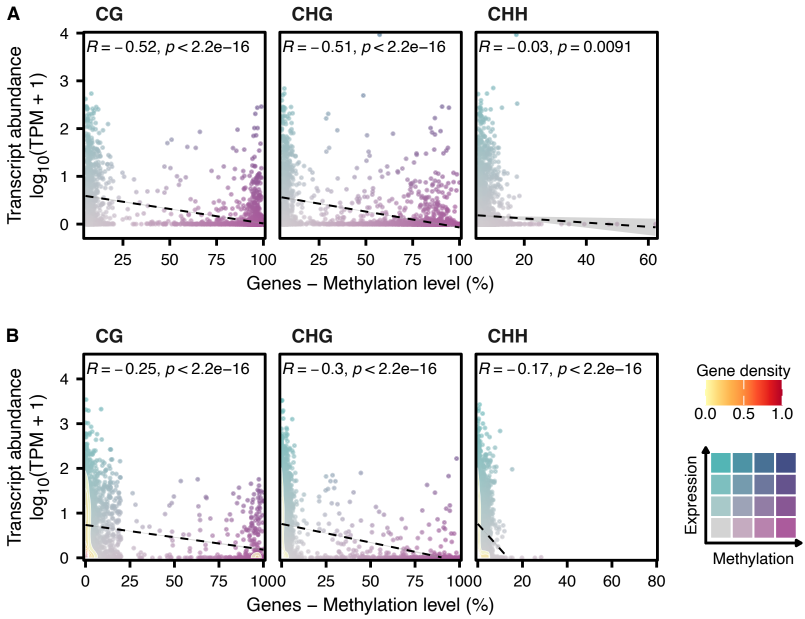

Figure S2. Gene expression relative to methylation level per cytosine context.
The methylation level (x-axis) is represented relative to the gene expression (y-axis) for TE-like genes (**A**) and genes not categorized as gbM or teM (**B**). For visual representation, points were randomly subsampled to 10,000 for **B**. gbM: gene body methylation, teM: TE-like methylation.

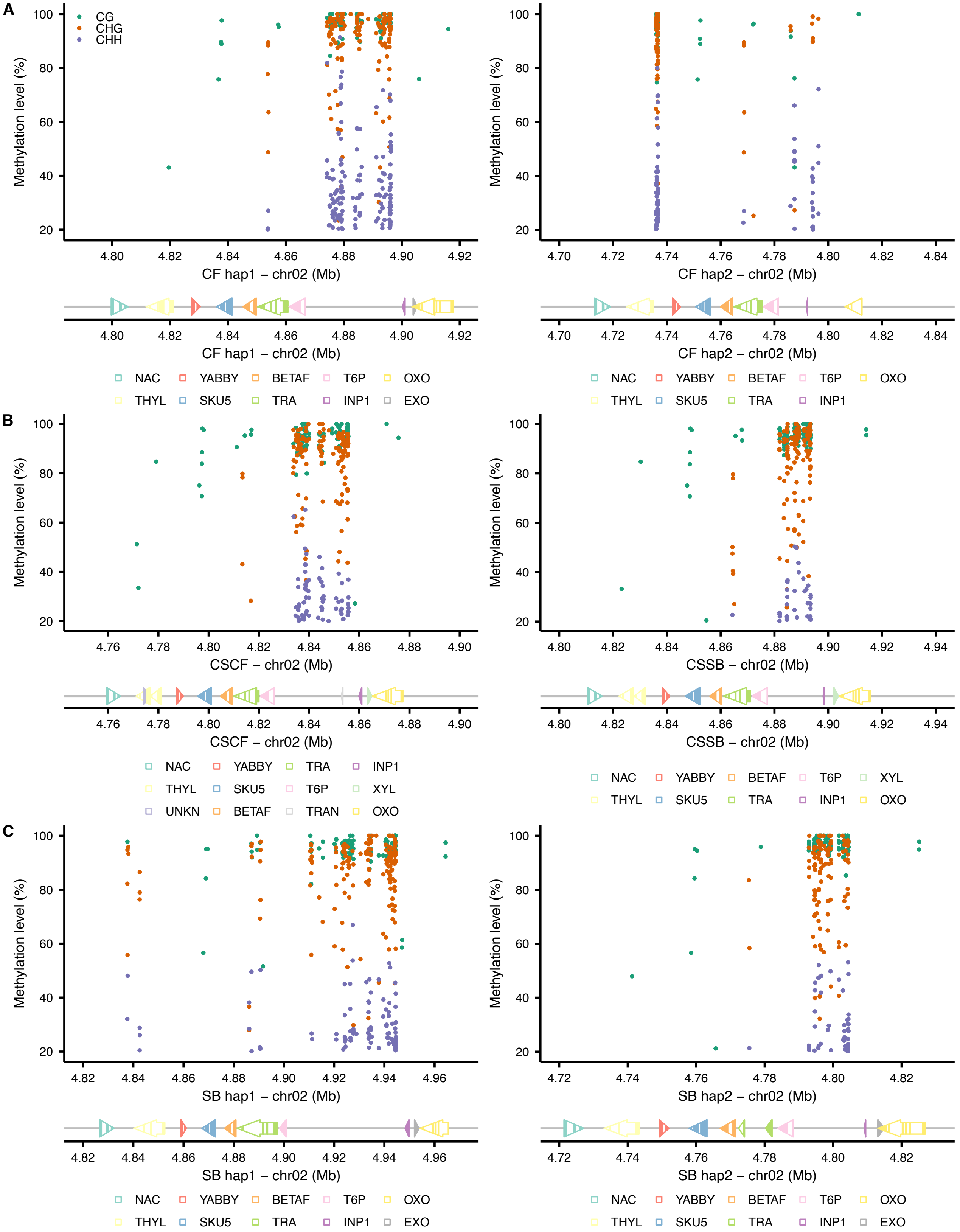

Figure S3. Methylation patterns are inherited at the sex-determining region.
On each plot, the top panel represents the methylation levels (filtered to be at least 20%) and the bottom panel represents the gene annotations for **A**, Cabernet Franc (hap1 on the left, hap2 on the right); **B**, Cabernet Sauvignon (hapCF on the left, hapSB on the right); and **C**, Sauvignon Blanc (hap1 on the left, hap2 on the right).

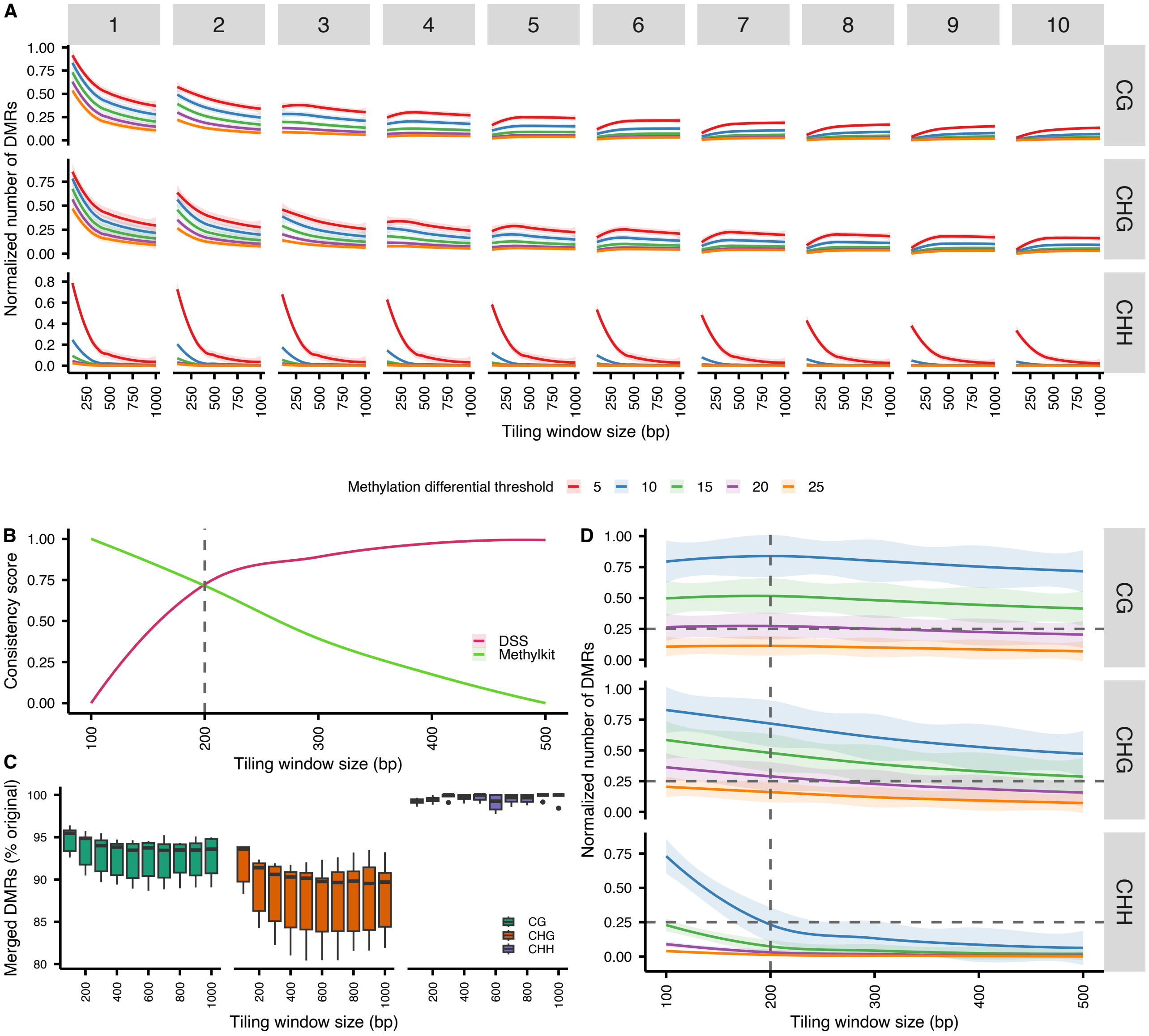

Figure S4. Benchmark for differentially methylated regions (DMRs).

A. Results of methylkit differential methylation analyses normalized per context (3 comparisons x 2 references, n = 6). Each column represents a minimum coverage threshold. **B**. Consistency scores based on the number of DMRs detected in common between methylkit and DSS for the context CG (3 comparisons x 2 references x 2 differential directions x 4 differential thresholds, n = 48). The dashed line represents the tiling window size with the highest consistency score. **C**. Number of DMRs represented as a percentage of original DMRs before merging adjacent genomic ranges (3 comparisons x 2 references, n = 6). The middle bars represent the median while the bottom and top of each box represent the 25th and 75th percentiles, respectively. The whiskers extend to 1.5 times the interquartile range and data beyond the end of the whiskers are plotted individually as outlying points. **D**. Final selection of the tiling window size and methylation differential thresholds per context (3 comparisons x 2 references, n = 6). The point of intersection between the horizontal and the vertical dashed lines indicate the parameter selected per context. For **A**, **B**, and **D**, the lines represent smoothed conditional means with a 0.95 confidence interval.

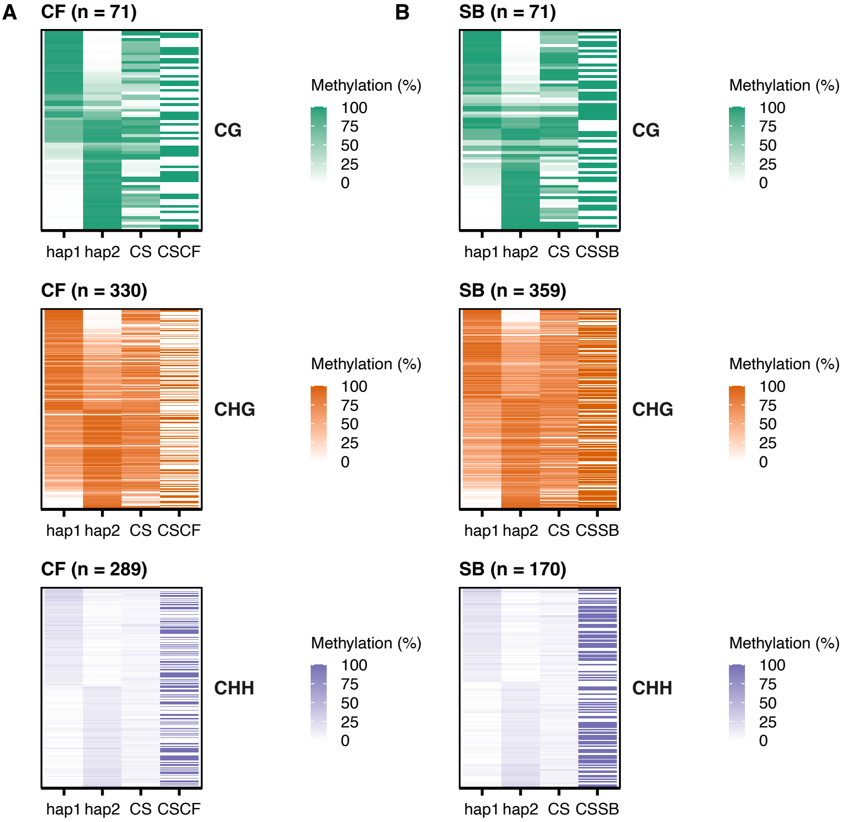

Figure S5. Methylation levels in the intra-cultivar DMRs.
Methylation levels in the regions defined as DMR for the intra-cultivar DMA performed (hap1 vs hap2) in CF (**A**) and SB (**B**). CS methylation levels are represented in the third column. Values are displayed by descending order of methylation differential per cytosine context. Colors in the last column represent the presence in the corresponding haplotype (**A**, CSCF; **B**, CSSB), white color indicates presence in the alternative haplotype (**A**, CSSB; **B**, CSCF).

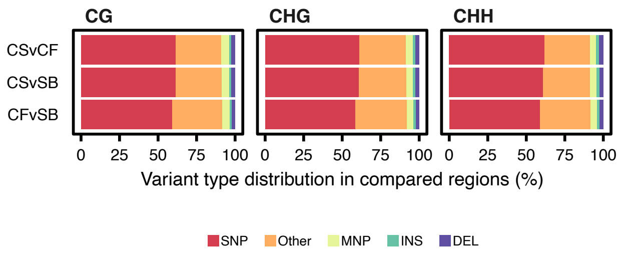

Figure S6. Distribution of the graph-inferred variants in the DMRs.
After converting the sequence graph into a vcf, the variants were overlapped with the DMRs.

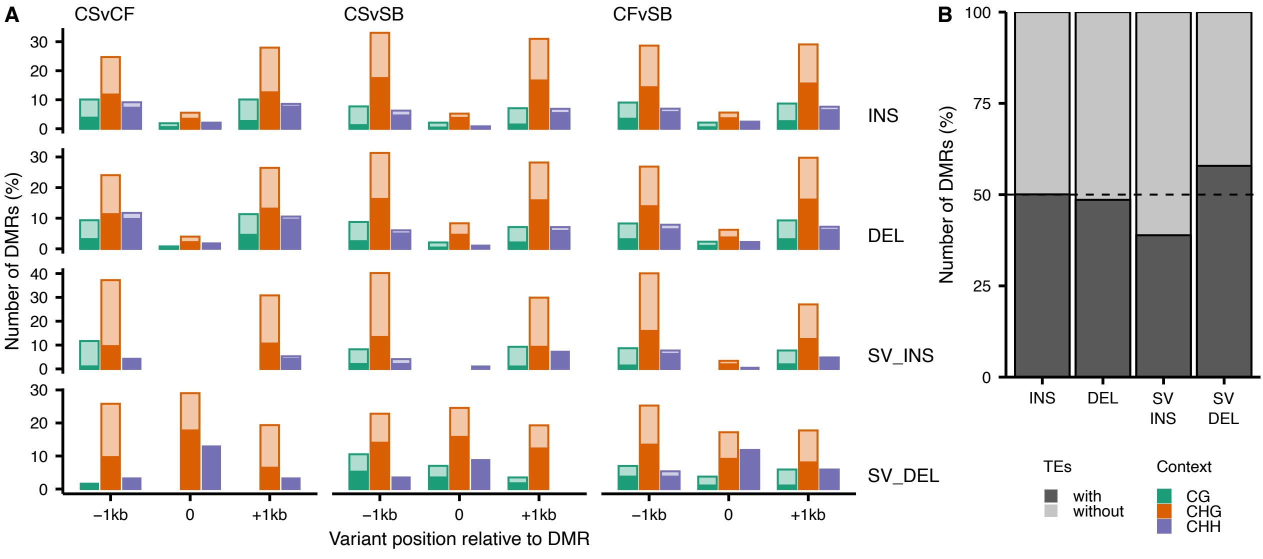

Figure S7. Distribution of the variant types relative to their distance from DMRs.

A. For each panel, the number of DMRs is represented as a proportion of the total number of DMRs in the panel. Any variant detected upstream a DMR up to 1 kb is included in the '-1 kb' x-axis, variants occuring within a DMR have a relative position of 0, and variants downstream the DMR up to 1 kb are included in the '1 kb' x-axis. For each bar, the transparency levels represent the percentage of DMRs with TEs. INDELs with a length greater than or equal to 50 bp are classed as structural variants (SV). **B.** Overall proportion of DMRs impacted by variants overlapping TEs.

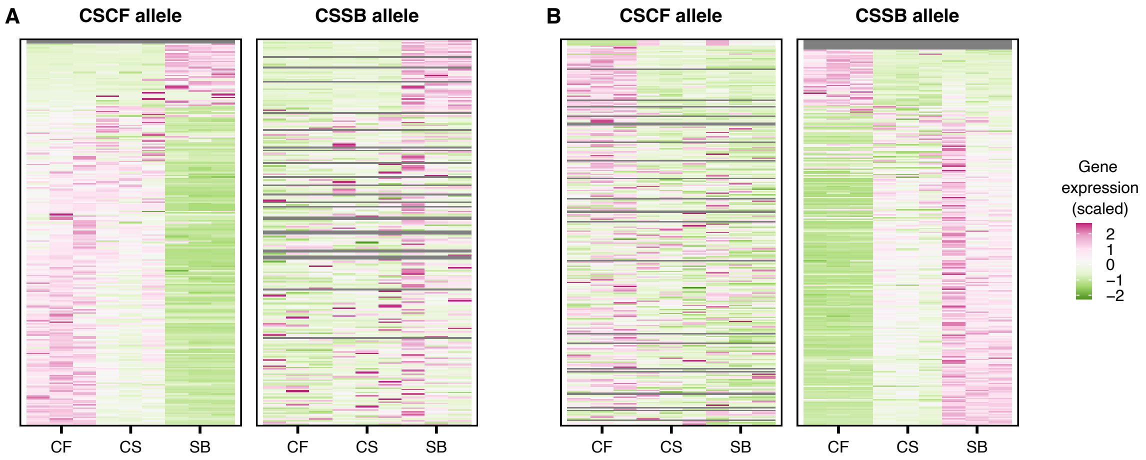

Figure S8. Allelic expression of the iCF and iSB gene sets.

A. iCF gene set: for each allelic pair, the expression of the CSCF allele and the CSSB allele is represented on the left and the right panel, respectively. Each row corresponds to a gene, each column represents a sample. **B.** iSB get set. Gene expression values were scaled per gene and per allele represented as a color gradient from green (low expression) to pink (high expression). The grey color indicates the absence of count data for the allele.

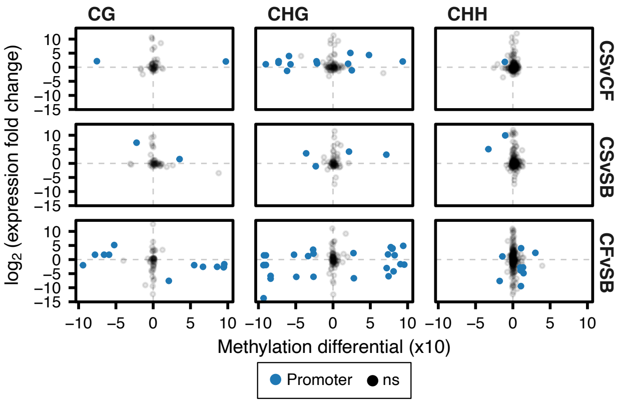

Figure S9. Gene expression relative to promoter methylation level per cytosine context.
Comparison of the methylation differential to the expression fold changes per cytosine context and comparison in the promoters. ns: not significant.

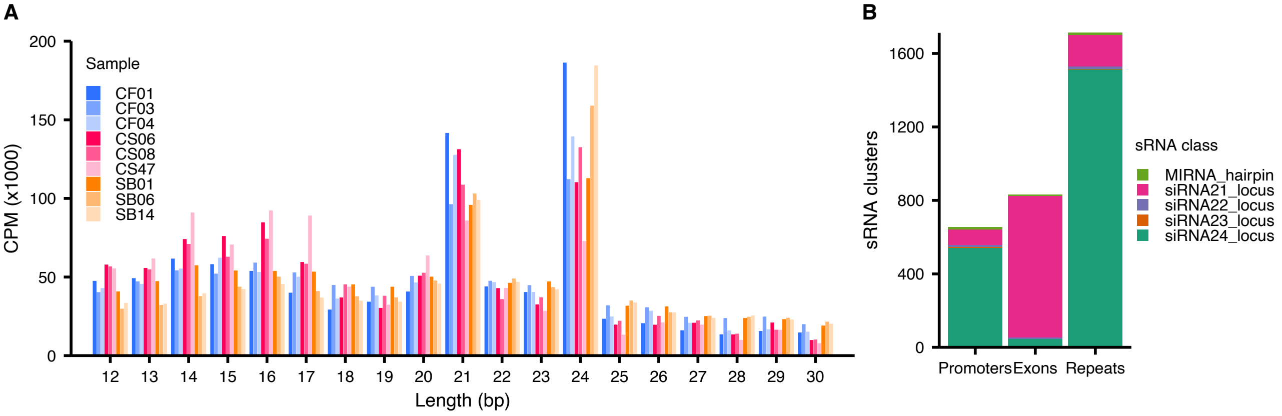

Figure S10. Distribution of the sRNAs in the genome of CS.

A. Size distribution of the loci detected by ShortStack in the genome of CS for each leaf sample. Data are represented as CPM (counts per million mapped reads) for sequences ranging from 12 to 30 bp. For visual representation, extreme count outliers with a frequency z-score >= 25 were discarded (0.00042% of the dataset). **B.** sRNA in gene and repeat annotation. sRNA were considered if the overlap with the feature was greater than or equal to 80%.

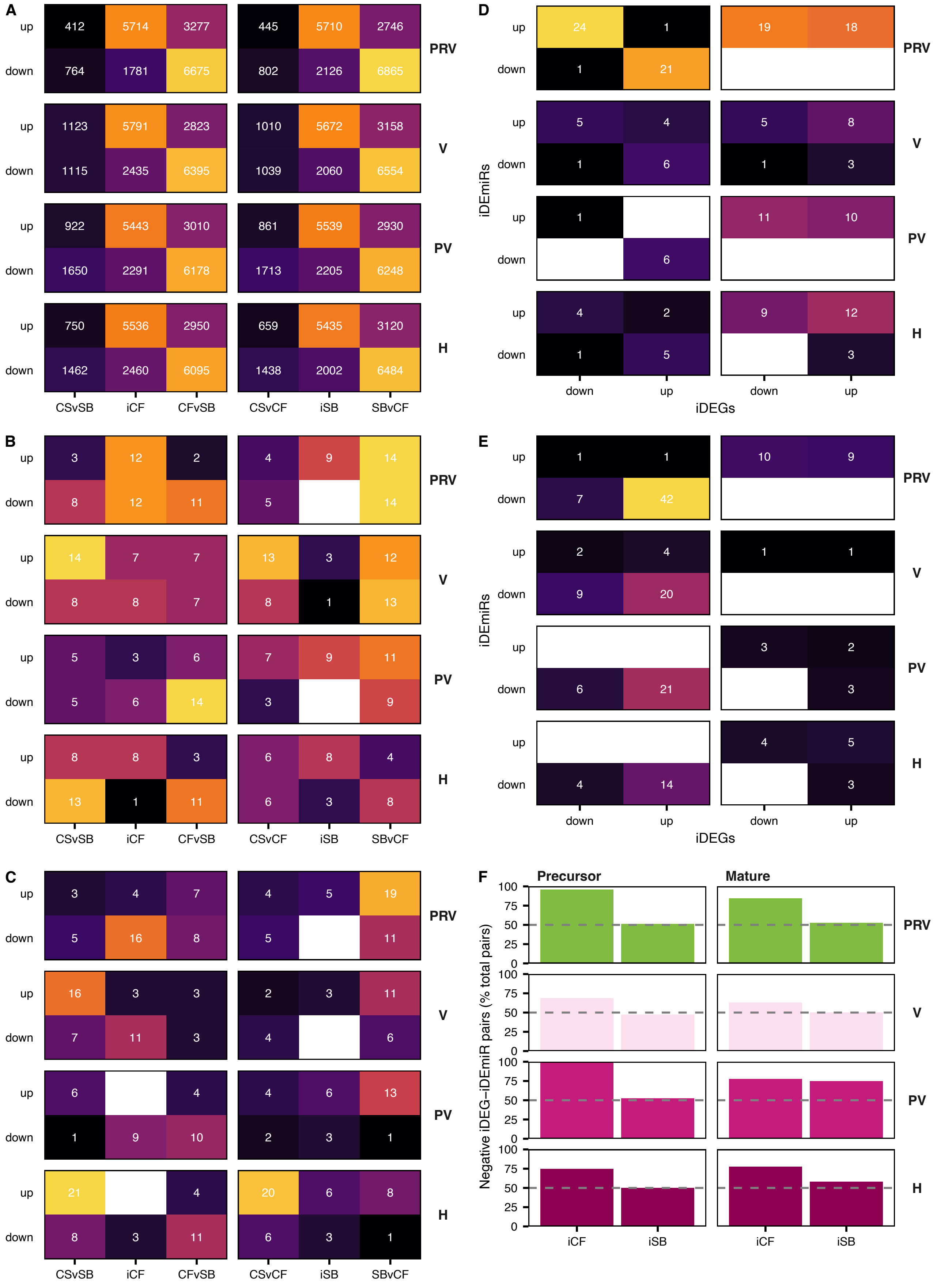

Figure S11. Identification of iDEGs and iDEmiRs in berries.

A. Differential expression analysis (DEA) results for the genes at four different berry ripening stages: PRV, pre-veraison; V, veraison; PV, post-veraison, and H, harvesting. On the left, CSvSB and CFvSB are compared, the intersection defines iCF. On the right, CSvCF and SBvCF are compared, the intersection defines iSB. DEA for miRNA precursors (**B**) and mature miRNAs (**C**). **D.** Intersection for the miRNA precursors between the iDEGs defined in (**A**) and the iDEmiRs defined in (**B**). **E.** Intersection for the mature miRNAs between the iDEGs defined in (**A**) and the iDEmiRs defined in (**C**). **F.** Proportion of negative pairs (up-regulated vs down-regulated) between iDEGs and iDEmiRs for miRNA precursors (left) and mature miRNAs (right). The dashed line represents 50%.
